# Epitope mirroring between the malaria surface proteins PfGARP and PIESP2 identifies a knob-associated complex in infected erythrocytes

**DOI:** 10.1016/j.jbc.2026.113291

**Published:** 2026-06-23

**Authors:** Christopher J. Schwake, Rachel M. Krueger, Feier Chen, Dalia Karim, Madeline J. Mueller, Sanjay A. Desai, Josh R. Beck, Toshihiko Hanada, Athar H. Chishti

**Affiliations:** 1 Program in Cellular, Molecular, and Developmental Biology, Graduate School of Biomedical Sciences, Tufts University School of Medicine, Boston, Massachusetts, USA; 2 Department of Developmental, Molecular, and Chemical Biology, Tufts University School of Medicine, Boston, Massachusetts, USA; 3 Program in Pharmacology and Drug Development, Graduate School of Biomedical Sciences, Tufts University School of Medicine, Boston, Massachusetts, USA; 4 Laboratory of Malaria and Vector Research, National Institute of Allergy and Infectious Diseases, National Institutes of Health, Rockville, Maryland, USA; 5 Department of Biomedical Sciences, Iowa State University, Ames, Iowa, USA; 6 Program in Molecular Microbiology, Graduate School of Biomedical Sciences, Tufts University School of Medicine, Boston, Massachusetts, USA

**Keywords:** cytoadhesion, knob, PfGARP, PIESP2Band 3, *Plasmodium falciparum*

## Abstract

Malaria is a life-threatening infectious disease responsible for an estimated 610,000 deaths each year, with the greatest burden falling on children in sub-Saharan Africa. A defining feature of infection by the most lethal human malaria parasite, *Plasmodium falciparum*, is the adhesion of infected red blood cells (iRBCs) to vascular endothelial cells. This cytoadhesion is mediated by distinctive knob-like protrusions unique to *P*. *falciparum* and plays a central role in the pathogenesis of cerebral and placental malaria, contributing to seizures, coma, and adverse pregnancy outcomes. Despite decades of intensive efforts to target highly polymorphic PfEMP1 anchored on knobs, no effective therapeutic interventions targeting this pathway have yet been developed. Using phage display cDNA technology, we previously identified a malaria antigen called *P*. *falciparum* glutamic acid-rich protein (PfGARP), which binds to host erythrocyte membrane band 3 (SLC4A1). To further investigate the composition of the PfGARP complex, we generated and characterized a monoclonal antibody, termed GM7mAb, that recognizes an epitope located within the repetitive regions of PfGARP. By combining unbiased mass spectrometry and two independent PfGARP-null parasite lines, here we report the identification of a reverse epitope recognized by GM7mAb within Parasite-Infected Erythrocyte Specific Protein-2 (PIESP2). These findings demonstrate the presence of a subpopulation of PIESP2 localized to the surface of knobs, and the utility of GM7mAb as a diagnostic tool for the detection of antibodies against PIESP2 in a malaria-endemic region. Further characterization of the knob-associated PIESP2 complex may yield novel insights into strategies for disrupting cytoadherence in cerebral and pregnancy-associated malaria.

Malaria remains a devastating parasitic disease that causes substantial mortality in endemic regions, with an estimated 610,000 deaths annually (2024). Combination therapies consisting of artemisinin derivatives, quinine-based compounds, and partner drugs continue to represent the most effective treatment options. However, the emergence and spread of resistance to anti-malarial drugs pose a serious and growing threat. Although recent advances in malaria vaccine development are encouraging (1, 2), no vaccine currently provides sufficiently broad and durable protection for large-scale immunization programs in developing countries. Therefore, the identification of novel therapeutic targets and the development of new anti-malarial vaccines remain urgent priorities.

*Plasmodium falciparum* is the most lethal of the five *Plasmodium* species known to infect humans. Mortality associated with *P*. *falciparum* infection is largely attributable to its unique ability to mediate cytoadherence, whereby infected red blood cells (iRBCs) attach to the vascular endothelium of post-capillary venules, leading to severe disease manifestations (3). During intraerythrocytic development, the parasite exports more than 400 proteins that traverse multiple membrane compartments within the iRBC. One of the most prominent and unique structural modifications in iRBCs is the formation of electron-dense surface protrusions known as “knobs” that facilitate cytoadherence and account for over 90% of malaria-related mortality (3, 4).

A key parasite-derived protein localized to knobs is *P*. *falciparum* erythrocyte membrane protein 1 (PfEMP1) (5, 6, 7). PfEMP1 is a single-pass type 1 transmembrane protein encoded by approximately 60 highly diverse members of the var gene family. The assembly and maturation of knobs involve both parasite and host erythrocyte proteins, which collectively influence the adhesive phenotype mediated by PfEMP1. Each PfEMP1 variant contains a highly polymorphic extracellular domain composed of defined subdomains that interact with specific endothelial receptors, thereby contributing to the severe clinical sequelae of malaria (3). Despite its central role in severe malaria pathogenesis, the extensive polymorphism of PfEMP1 has hindered the development of targeted therapeutic interventions against the cytoadherence pathway. Although the molecular mechanisms underlying cerebral and pregnancy-associated malaria (PAM) are complex, it is widely accepted that disrupting interactions between knob-associated PfEMP1 and endothelial receptors represents a promising strategy for attenuating virulence in severe malaria.

The application of multiple phage display cDNA screens using intact red blood cells (RBCs) as bait; previously, we identified *P*. *falciparum* glutamic acid-rich protein (PfGARP) as a binding partner of human erythrocyte band 3 (anion exchange protein 1, AE1, SLC4A1) (8). PfGARP (PF3D7_0113000, PFA0620c) is expressed during the trophozoite and schizont stages of intraerythrocytic parasite development, coinciding with knob formation and cytoadhesion. Its expression is restricted to *P*. *falciparum*, and antibodies against PfGARP have been detected in children resistant to malaria (8, 9, 10). In contrast to the *var* gene family, which encodes approximately 60 antigenically distinct variants of PfEMP1, PfGARP is encoded by a single conserved gene on chromosome 1 with no detectable sequence variation in the parasite genome. An independent study generated a monoclonal antibody (mAb7899) through a commercial facility and defined a specific linear epitope in PfGARP (VKNVIEDEDKDGVEIIN) (10). The mAb7899 was reported to induce parasite death in infected RBCs (iRBCs) *in vitro* and partially reduced infection in a nonhuman primate malaria model *in vivo* (10). These findings suggest that PfGARP may play a dual functional role in adhesion and cell death pathways (8, 10).

The precise role of PfGARP in cytoadhesion remains unclear. It is not known whether PfGARP interacts with other parasite-derived proteins within iRBCs and no cognate receptor has been identified on host endothelial cells. To investigate the composition of the PfGARP-associated complex in iRBCs, we employed unbiased mass spectrometry to identify interacting proteins using a monoclonal antibody designated as GM7mAb that was generated in our laboratory (8). Here, we report the serendipitous identification of reverse epitopes in both PfGARP and PIESP2 (Parasite-Infected Erythrocyte Surface Protein 2; PF3D7_0501200, PFE60) that are recognized by GM7mAb. PIESP2 has been implicated in Maurer’s cleft reorganization, virulence protein export, and adhesion-related pathways in severe malaria (11, 12, 13, 14, 15, 16, 17, 18, 19). However, its direct role in cellular adhesion has not been established. Using the dual-specificity GM7mAb and immuno-electron microscopy, we provide the first evidence that a subset of PIESP2 localizes to surface knobs on iRBCs. Combined with multiple gene knockouts of PfGARP and PIESP2 and immunofluorescence microscopy, our findings reveal a previously unrecognized macromolecular complex in iRBCs composed of parasite-derived PIESP2 and PfGARP together with host band 3. These findings provide new mechanistic insights into the role of knobs in cytoadhesion during malaria pathogenesis.

## Results

### PfGARP phylogeny and sequence analysis

**Figure 1:**
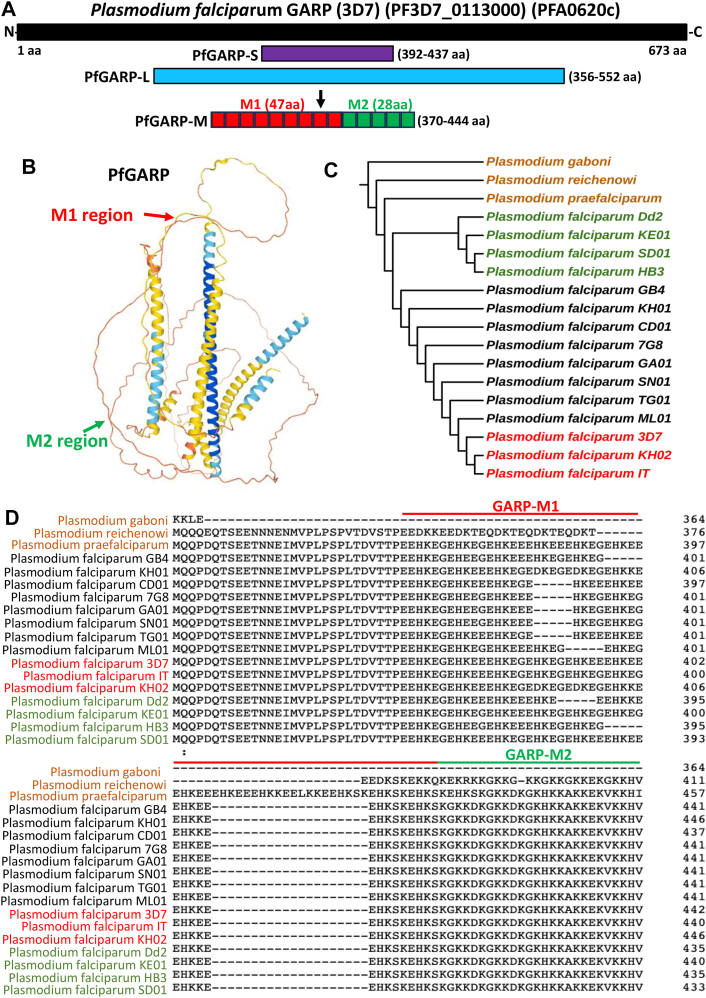
**PfGARP phylogeny and sequence analysis.***A*, schematic diagram of PfGARP (PF3D7_0113000) and expression constructs of specific segments. PfGARP consists of 673 amino acids. Multiple phage clones identified in our previous screens (8) were used to design expression constructs designated as PfGARP-S, PfGARP-L and PfGARP-M. PfGARP-M was designed to include both M1 and M2 regions encoding immunoreactive (*red color* repeats) and RBC-binding domain (*green* color repeats), respectively. *B*, structure prediction analysis of PfGARP using AlphaFold (AI-system, DeepMind). The M1 and M2 regions are marked by the *red* and *green arrows*. Details of the color-coded positions of the amino acids encoding the M1 and M2 regions of PfGARP are available in the current version of AlphaFold. *C*, phylogenetic analysis of PfGARP sequences deposited in PlasmoDB. *D*, alignment of PfGARP-M1 and M2 domain sequences deposited in PlasmoDB.

Using phage display cDNA libraries constructed from sialic acid-independent and dependent *P*. *falciparum* 3D7 and FCR3 strains, respectively, we initially characterized two phage clones corresponding to the middle of the *P*. *falciparum* glutamic acid-rich protein, PfGARP (PF3D7_0113000, PFA0620c) gene (8). Both untreated and neuraminidase-treated human erythrocytes were used as bait (8). The larger clone isolated from the FCR3 strain was designated as PfGARP-L, and the smaller clone was named as PfGARP-S (Fig. 1*A*) (8). Using structure-prediction analysis of the primary structure of both short and long clones, we designed a codon-optimized stable construct termed PfGARP-M (Fig. 1*A*) (8). The PfGARP-M clone contained two repetitive regions. The M1 segment composed of 47 amino acids contained the epitope recognized by GM7mAb and the 28 amino acids M2 segment mediated direct binding of PfGARP to human erythrocytes (Fig. 1, *A* and *B*) (8). A comprehensive sequence comparison of PfGARP homologues was performed, including *Plasmodium praefalciparum*, *Plasmodium reichenowi*, and *Plasmodium gaboni* by utilizing data from the PlasmoDB (Fig. 1, *C* and *D*). Sequence comparison revealed the closest phylogenetic relationship between the human *pfgarp* gene and its homolog in *P*. *praefalciparum*. *P*. *praefalciparum* is closest to two chimpanzee *Plasmodium* parasites showing the most distant relationship (Fig. 1, *C* and *D*). Notably, within *P*. *falciparum*, a monophyletic grouping was observed with isolates from the Dd2, KE01, SD01, and HB3 strains, while the commonly used 3D7 strain exhibited closest relatedness to the KH02 and IT strains. AlphaFold (20) structural prediction for PfGARP showed relatively weak predictive power, likely due to the non-homologous sequence feature of the gene (Fig. 1*B*). In all parasite strains examined, both the PfGARP-M1 and PfGARP-M2 regions demonstrated high conservation across all isolates (Fig. 1*D*). Interestingly, the M1 region recognized by GM7mAb displayed variability in repeat numbers and contained several single-nucleotide polymorphisms in the DNA sequences deposited in the PlasmoDB (SNPs). The variability of M1 region in the DNA sequences aligns with the high immunogenicity observed during the development of the GM7mAb (8). These observations suggest that selective evolutionary pressure may have contributed to the conservation of the RBC-binding property of the PfGARP-M2 domain (8). Interestingly, *P*. *gaboni* GARP is missing both the M1 and M2 regions (Fig. 1*D*) thus implying that PgGARP may not elicit M1-mediated immune response and does not recognize homologous host band 3 in the erythrocytes of infected chimpanzees.

### PfGARP self-associates through the M2 domain

**Figure 2:**
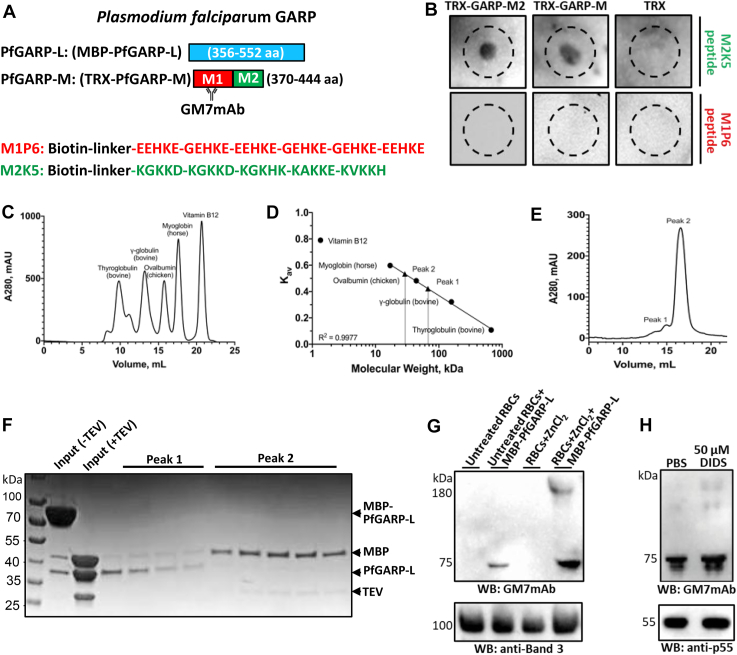
**Self-association of PfGARP.***A*, self-assembly of PfGARP is mediated by the M2K5 peptide located within the M2 region of PfGARP-M (Green color), whereas the immunodominant M1 region does not participate in the self-assembly process. *B*, dot blot binding analysis of biotin-conjugated PfGARP-M peptides (M2K5 and M1P6) with recombinant fusion proteins. Representative image from three independent experiments. *C*, determination of the oligomeric state of the PfGARP-L construct by size-exclusion chromatography (SEC). Gel filtration standards from Bio-Rad were used for calibration. *D*, SEC profile with protein standards. Respective positions of Peaks 1 and 2 derived from cleaved MBP-GARP-L. *E*, chromatogram of the cleaved Maltose Binding Protein (MBP)-PfGARP-L construct. *F*, SDS-PAGE analysis of peaks 1 and 2 following cleavage of the PfGARP-L construct. *G*, biochemical characterization of PfGARP-L binding to band 3 at the erythrocyte surface. PfGARP-L preferentially binds to clustered band 3 on the erythrocyte surface. *H*, DIDS incubation of erythrocytes does not inhibit PfGARP binding. Bands detected by the GM7mAb are shown here.

Our previous findings (8) demonstrated that PfGARP-M can induce RBC aggregation implying that PfGARP might achieve this aggregation activity through self-association, a phenomenon commonly observed in proteins with tandemly repetitive regions (21). To test this model, a dot immunoblotting assay was used to determine the self-association region within PfGARP-M segment. Using biotin-conjugated synthetic peptides designed from respective domains of PfGARP-M (Fig. 2*A*), dot blotting demonstrated that the M2 region of PfGARP is sufficient for PfGARP self-association. The M2K5 peptide recognized immobilized PfGARP-M2 as well as PfGARP-M segments (Fig. 2*B*, green color). In contrast, M1P6 peptide did not detect any region of immobilized PfGARP-M (Fig. 2*B*, red color). This finding was further confirmed by size-exclusion chromatography revealing a homo-oligomeric state of PfGARP-L migrating as a trimer or tetramer consistent with its self-association state under these conditions (Fig. 2, *C*–*F*). Previous studies by Sherman and colleagues (22, 23, 24, 25, 26) have reported the functional role of host band 3 aggregation in parasite adhesion. The pretreatment of erythrocytes with DIDS (4,4′-diisothiocyano-2,2′-stilbenedisulfonic acid) has been shown to block parasite-induced band 3 clustering (22, 24). Similarly, treatment with Zinc chloride has been established as a chemical modality for inducing band 3 clustering (22), mimicking the clustering observed in iRBCs. We treated human RBCs with zinc chloride and observed an increase in PfGARP binding as well as the formation of larger oligomers (Fig. 2*G*). These observations suggest that PfGARP forms oligomers upon binding to clustered band 3 molecules. To further investigate if the DIDS binding region (DBR) of band 3 participates in PfGARP binding, the RBCs were incubated with DIDS prior to incubation with MBP-GARP-L as described in the zinc chloride experiment. The DIDS treatment did not have any discernable effect on PfGARP binding (Fig. 2*H*). Similarly, treatment with zinc chloride followed by DIDS labeling did not alter PfGARP binding to RBCs (data not shown). Together, these findings begin to clarify the biochemical basis of PfGARP self-association and role of host band 3 clustering in inducing RBC aggregation by PfGARP (8).

### PfGARP interactome at the erythrocyte cell surface

**Figure 3:**
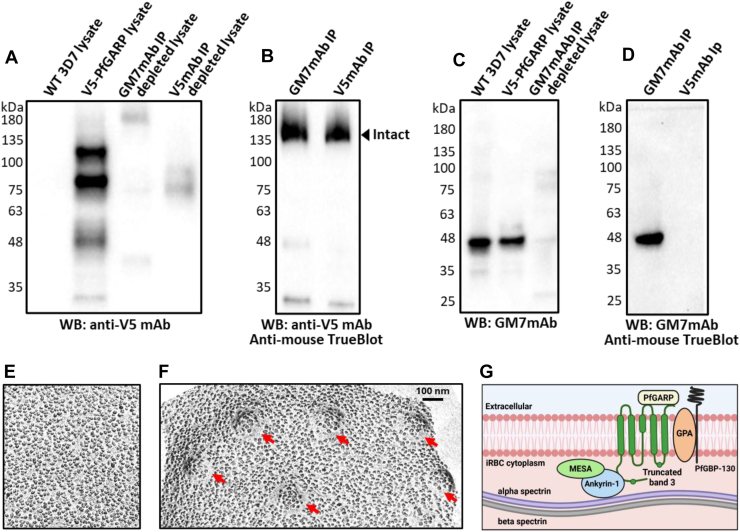
**Biochemical characterization of V5-PfGARP construct by GM7mAb and anti-V5 mAb.***A*, V5-PfGARP transfected parasite lysate was depleted with either GM7mAb or anti-V5mAb and supernatant was immunoblotted with anti-V5mAb. Multiple translation products of V-PfGARP were removed by these antibodies. *B*, to evaluate the nature of immunoprecipitated material, magnetic beads bound to GM7mAb and anti-V5mAb were analyzed for the presence of intact V5-PfGARP using anti-V5mAb and anti-mouse TrueBlot system. This panel shows only full-length V5-PfGARP after immunocapturing since this is a C-terminal V5 tag construct. Details of the intact bands detected are described in the text and Experimental procedure. *C*, *D* and *A* similar set of immunoblotting experiments were performed using GM7mAb combined with the anti-mouse TrueBlot system. The 48 kDa band was depleted by immunoprecipitation using both GM7mAb and anti-V5mAb. *E*, *F*, visualization of knobs by freeze-fracture electron microscopy. *Panel E* shows negative control using ring-infected erythrocytes, in which no knobs were observed. *Panel F* shows knob protrusions with intramembrane particles (IMPs) on the P-face of schizont-infected human erythrocytes. A clear circular zone surrounds the IMP clusters located on the knobs and across the remaining erythrocyte membrane. These IMPs likely represent clusters of band 3 and glycophorin A proteins. *G*, schematic summary of V5-PfGARP complex in the iRBCs. GBP-130, Glycophorin binding protein-130 (precise membrane orientation GBP-130 is not known); GPA, Glycophorin A; MESA, Mature parasite-infected erythrocyte surface antigen. IP-mass spectrometry data are shown in Tables 1 and 2.

To evaluate the composition of PfGARP complex, the transfected V5 tagged PfGARP construct was utilized (10, 27). Immunoprecipitation of V5-PfGARP with anti-V5 mAb was followed by unbiased mass spectrometry to identify interacting partners of PfGARP. Using anti-V5 mAb immunoblotting, we first confirmed that both GM7mAb and anti-V5 mAb can deplete V5 tagged PfGARP from the transfected parasite lysates (Fig. 3*A*). Similarly, immunoblotting using the anti-V5 mAb immunoblotting (Mouse TrueBlot) identified a stable form of V5-PfGARP of ∼165 kDa in the immunoprecipitated material by both GM7mAb and anti-V5 mAb (Fig. 3*B*). Of note, we used the same V5 tagged PfGARP expression system that has been described in a previous study (10). The molecular weight of V5-tagged PfGARP protein was estimated as ∼124 kDa in the previous study (10). However, the apparent mobility of V5-tagged PfGARP fusion protein determined by SDS-PAGE is ∼165 kDa owing to its highly acidic amino acid composition in the previous study (10). Next, we evaluated the status of 48 kDa band by immunoblotting using GM7mAb in the lysates of wild-type 3D7 and V5-PfGARP lines as well as GM7mAb-depleted parasite lysate (Fig. 3*C*). As expected, the 48 kDa was removed in the parasite lysate upon depletion with GM7mAb (Fig. 3*C*). This observation was confirmed by the presence of 48 kDa band in the GM7mAb-immunoprecipitated material but not detected in the anti-V5 mAb-immunoprecipitated material by GM7mAb (Mouse TrueBlot) immunoblotting (Fig. 3*D*).

Using the same pulldown strategy, we performed unbiased mass spectrometry (Harvard Medical School Core Facility) to identify the interacting partners of V5-PfGARP by testing magnetic beads coated with anti-V5 mAb (Table 1). Late-stage *P*. *falciparum* 3D7 parasites transfected with V5-PfGARP were harvested, solubilized in RIPA buffer, and immunoprecipitated with anti-V5 mAb antibody in two independent experiments (Table 1). We identified 37 different proteins associated with V5-PfGARP interactome, considering hits positive only if there were at least 2 unique peptides from each independent experiment (Table 1). The most abundant proteins included alpha and beta polypeptides of host spectrin, PfGARP, mature-parasite-infected erythrocyte surface antigen (MESA/PF3D7_0500800), host ankyrin-1, and parasite glycophorin binding protein (GBP-130/PF3D7_1016300). Importantly, host band 3 (anion exchanger AE1/SLC4A1) was also identified as an interacting partner, confirming our previous findings showing band 3 as a potential host receptor for PfGARP (8). To further corroborate the presence of band 3 in knobs of iRBCs, we visualized the distribution of intramembrane particles (IMP), which are mainly composed of band 3 (28), in *P*. *falciparum* infected human RBCs by freeze-fracture electron microscopy in both knobless and knobby 3D7 strains, respectively (Fig. 3, *E* and *F*). The IMPs were clustered on knobs, showing a hollow ring-like zone surrounding the periphery of knobs (Fig. 3*F*, red arrows). The major interactions identified by IP-mass spectrometry of V5-PfGARP complex immunoprecipitated by anti-V5 mAb are summarized (Fig. 3*G*). A similar IP-mass spectrometry analysis was performed to validate the composition of V5-PfGARP complex immunoprecipitated with GM7mAb (Table 2). PfGARP was identified as a top peptide hit, thus confirming that GM7mAb can detect endogenous V5-PfGARP. Of note, both V5-PfGARP and endogenous PfGARP will be pulled down by GM7mAb under these conditions. Several parasite and host proteins were also identified by this screen (Table 2).

**Table 1:** Identification of interacting partners of V5-PfGARP *via* immunoprecipitation with ant-V5 mAb and mass spectrometry analysis

Number	Gene symbol	Annotation	Host/parasite	MW (kDa)	Unique V5-1	Total V5-1	Unique V5-2	Total V5-2
1	SPTA1	Spectrin alpha chain	Host	280	19	19	18	19
2	SPTB	Spectrin beta chain	Host	246	13	13	16	16
3	PF3D7_0113000	Glutamic acid-rich protein	Parasite	80	14	32	15	46
4	PF3D7_0500800	Mature parasite-infected erythrocyte surface antigen	Parasite	168	13	15	13	14
5	ANK1	Ankyrin-1	Host	206	13	14	14	16
6	PF3D7_0507100	60S ribosomal protein	Parasite	46	7	7	6	7
7	PF3D7_1342000	40S ribosomal protein	Parasite	35	7	8	8	9
8	PF3D7_1016300	Glycophorin binding protein	Parasite	96	6	8	6	7
9	PF3D7_0831700	Heat shock protein 70	Parasite	75	5	5	4	5
10	HBB	Hemoglobin subunit beta	Host	16	3	3	6	6
11	PF3D7_0917900	Heat shock protein 70	Parasite	72	3	3	6	6
12	PF3D7_1408600	40S ribosomal protein	Parasite	25	4	4	4	4
13	PF3D7_1027300	Peroxiredoxin	Parasite	44	4	4	3	3
14	PF3D7_0818900	Heat shock protein 70	Parasite	74	2	2	2	2
15	PF3D7_1011800	PRE-binding protein	Parasite	132	3	3	3	3
16	TUBA1C	Tubulin alpha-1C	Host	50	3	4	3	3
17	PF3D7_1105000	Histone H4	Parasite	12	4	4	5	6
18	SLC4A1	Band 3 anion transport protein	Host	101	4	4	5	5
19	ANXA2	Annexin A2	Host	39	4	4	4	5
20	PRDX2	Peroxiredoxin-2	Host	22	3	3	4	4
21	PF3D7_1424100	60S ribosomal protein L5, putative	Parasite	34	2	2	3	3
22	PF3D7_0708400	Heat shock protein 90	Parasite	86	3	3	4	4
23	PF3D7_0614500	60S ribosomal protein L19	Parasite	22	2	2	2	2
24	PF3D7_1424400	60S ribosomal protein L7-3	Parasite	33	2	2	3	3
25	PF3D7_0503300	Serine/arginine-rich splicing factor 12	Parasite	38	2	2	2	2
26	CPK1	Calcium-dependent protein kinase 1	Host	61	2	2	4	4
27	HSPA5	78 kDa glucose-regulated protein	Host	72	2	2	2	3
28	PF3D7_0813900	40S ribosomal protein S16, putative	Parasite	16	3	3	3	3
29	PF3D7_0818200	14-3-3 protein	Parasite	30	2	2	3	3
30	PF3D7_0610400	Histone H3	Parasite	15	3	3	2	3
31	PF3D7_0415900	Ribosomal protein L15	Parasite	24	2	2	2	2
32	HBA1	Hemoglobin subunit alpha	Host	15	2	2	2	2
33	PF3D7_1224000	GTP cyclohydrolase I	Parasite	46	2	2	2	2
34	PF3D7_0401800	Uncharacterized protein	Parasite	60	3	3	2	2
35	PF3D7_0903700	Tubulin alpha chain	Parasite	50	2	3	2	2
36	PF3D7_1460700	60S ribosomal protein L27	Parasite	17	2	2	2	2
37	PF3D7_0320900	Histone H2A	Parasite	16	2	2	2	2

**Table 2:** Identification of interacting partners of V5-PfGARP *via* immunoprecipitation with GM7mAb and mass spectrometry analysis

Number	Gene symbol	Annotation	Host/parasite	MW (kDa)	Unique GM7-1	Total GM7-1	Unique GM7-2	Total GM7-2
1	PF3D7_0113000	Glutamic acid-rich protein	Parasite	79	19	43	14	26
2	PF3D7_0501200	Parasite-infected erythrocyte surface protein	Parasite	48	19	50	10	28
3	PF3D7_1142100	Uncharacterized protein	Parasite	324	43	48	29	31
4	PF3D7_0730900	EMP1-trafficking protein	Parasite	244	44	63	25	37
5	PF3D7_1218500	Uncharacterized protein	Parasite	122	38	43	26	27
6	PF3D7_1149000	Antigen 332, DBL-like protein	Parasite	688	30	32	19	19
7	pdx1	Pyridoxal 5′-phosphate synthase subunit	Parasite	33	28	71	19	36
8	SPTA1	Spectrin alpha chain	Host	280	11	12	3	3
9	SPTB	Spectrin beta chain	Host	246	10	10	5	5
10	PF3D7_0500800	Mature parasite-infected erythrocyte surface antigen	Parasite	168	11	11	7	7
11	PF3D7_0815800	Vacuolar protein sorting-associated protein 9	Parasite	214	18	18	12	12
12	PF3D7_1252100	Rhoptry neck protein 3	Parasite	263	12	13	10	10
13	ANK1	Ankyrin-1	Host	206	12	12	8	8
14	PF3D7_0722200	Rhoptry-associated leucine zipper-like protein 1	Parasite	88	14	17	6	7
15	PF3D7_0510100	Uncharacterized protein	Parasite	297	13	13	4	4
16	PF3D7_0507100	60S ribosomal protein L4	Parasite	46	10	10	7	7
17	PF3D7_1228800	WD repeat-containing protein, putative	Parasite	379	11	11	5	6
18	PF3D7_1308400	Uncharacterized protein	Parasite	737	11	12	6	6
19	PF3D7_1342000	40S ribosomal protein S6	Parasite	35	9	14	8	9
20	PF3D7_0303200	HAD superfamily protein, putative	Parasite	135	9	9	6	6
21	PF3D7_1443000	Serine/threonine protein kinase	Parasite	150	9	9	4	4
22	PF3D7_1016300	Glycophorin binding protein	Parasite	96	4	4	4	4
23	PF3D7_1468100	Uncharacterized protein	Parasite	296	7	7	4	4
24	PF3D7_0831700	Heat shock protein 70	Parasite	75	7	8	5	5
25	PF3D7_1357000	Elongation factor 1-alpha	Parasite	49	7	7	2	2
26	MPP1	55 kDa erythrocyte membrane protein	Host	52	7	7	4	4
27	PF3D7_1406200	Uncharacterized protein	Parasite	338	4	4	4	4
28	PF3D7_0910200	Uncharacterized protein	Parasite	368	6	6	2	2
29	PF3D7_0715200	Uncharacterized protein	Parasite	258	5	5	2	2
30	SLC4A1	Band 3 anion transport protein	Host	102	4	5	4	4
31	PF3D7_0707800	RAP protein, putative	Parasite	142	4	4	2	2
32	PF3D7_1105100	Histone H2B	Parasite	13	4	4	4	4
33	ANXA2	Annexin A2	Host	39	4	4	4	5
34	PF3D7_0221700	Uncharacterized protein	Parasite	58	3	3	3	3
35	PF3D7_1465900	40S ribosomal protein S3	Parasite	25	4	4	4	5
36	PF3D7_0501000	Uncharacterized protein	Parasite	31	3	3	3	3
37	PF3D7_1016400	Serine/threonine protein kinase, FIKK family	Parasite	72	2	2	2	2
38	PF3D7_1370300	Membrane associated histidine-rich protein	Parasite	29	2	3	2	2
39	PFL2215w	Actin-1	Parasite	42	2	2	2	2

Finally, we performed similar IP-mass spectrometry screens on two independent PfGARP knockout parasite lines generated at Harvard Medical School (HMS) and National Institutes of Health (NIH), respectively (Table 3). The HMS PfGARP knockout line was originally generated by Dr Dvorin at HMS and has been previously characterized (10, 27). The second PfGARP knockout line was generated by our collaborator Dr Desai at NIH. The technical details of NIH PfGARP knockout construct are described (Supporting Fig. S1). PfGARP and host band 3 were identified as top hits in the wild-type 3D7 line immunoprecipitated by GM7mAb (Table 3). No PfGARP peptides were identified in the PfGARP knockout parasites (Table 3). Since this screen was performed using wild-type 3D7 parasite lysate providing further confirmation that GM7mAb can detect endogenous PfGARP under these conditions. The absence of band 3, spectrin, ankyrin and several parasite proteins in the PfGARP knockout parasites provides further credence to our proposed model (Fig. 3*G*) supporting the existence of a novel PfGARP macromolecular complex in the *P*. *falciparum* infected erythrocyte membrane.

**Table 3:** Validation of PfGARP KO through immunoprecipitation with GM7mAb and mass spectrometry analysis

Number	Gene symbol	Annotation	Host/parasite	MW (kDa)	Unique WT	Total WT	Unique KO	Total KO
1	PF3D7_0113000	Glutamic acid rich protein	Parasite	80	3	3	0	0
2	SLC4A1	Band 3 anion transport protein	Host	101	10	14	0	0
3	SPTA1	Spectrin alpha chain	Host	280	38	40	0	0
4	SPTB	Spectrin beta chain	Host	246	29	30	0	0
5	ANK1	Ankyrin-1	Host	206	24	27	0	0
6	PF3D7_1252100	Rhoptry neck protein 3	Parasite	263	24	26	0	0
7	PF3D7_1357000	Elongation factor 1-alpha	Parasite	49	9	11	0	0
8	PF3D7_0501200	Parasite-infected erythrocyte surface protein	Parasite	49	10	12	15	68
9	PF3D7_1462800	Glyceraldehyde-3-phosphate dehydrogenase	Parasite	37	7	8	0	0
10	PF3D7_0722200	Rhoptry-associated leucine zipper-like protein 1	Parasite	88	6	6	0	0
11	PF3D7_1343000	Phosphoethanolamine N-methyltransferase	Parasite	31	5	6	0	0
12	PF3D7_1222300	Endoplasmin, putative	Parasite	95	5	5	0	0
13	PF3D7_0202400	Gamete antigen 27/25, putative	Parasite	142	5	5	0	0
14	PF3D7_0202000	Knob-associated histidine-rich protein	Parasite	71	4	5	0	0
15	ACTG1	Actin, cytoplasmic 2	Host	42	4	5	0	0
16	PF3D7_0708400	Heat shock protein 90	Parasite	86	4	5	0	0
17	PFL2215w	Actin-1	Parasite	42	4	5	0	0
18	PF3D7_0929400	High molecular weight rhoptry protein 2	Parasite	163	4	5	0	0
19	PF3D7_1105000	Histone H4	Parasite	12	4	5	0	0
20	PF3D7_0500800	Mature parasite-infected erythrocyte surface antigen	Parasite	168	4	4	0	0
21	PF3D7_1324900	L-lactate dehydrogenase	Parasite	34	4	4	0	0
22	PF3D7_0917900	Heat shock protein 70	Parasite	72	4	4	0	0
23	PF3D7_0818200	14-3-3 protein	Parasite	30	4	4	0	0
24	PF3D7_1116800	Heat shock protein 101	Parasite	103	4	4	0	0
25	STOM	Erythrocyte band 7 integral membrane protein	Host	32	3	5	0	0
26	PF3D7_1424100	60S ribosomal protein L5, putative	Parasite	34	3	4	0	0
27	PF3D7_1228600	Merozoite surface protein 9	Parasite	87	3	3	0	0
28	PF3D7_0220000	Liver stage antigen 3	Parasite	176	3	3	0	0
29	MAL3P7.35	40S ribosomal protein S3a	Parasite	30	3	3	0	0

### Identification of PIESP2 antigen by GM7mAb

The unexpected findings from the GM7mAb immunoprecipitation-mass spectrometry (IP-MS) analysis of wild-type 3D7, V5-PfGARP transfected cells, and PfGARP knockout parasite lines (Tables 2 and 3) led us to investigate PIESP2 (**P**arasite-**I**nfected **E**rythrocyte **S**pecific **P**rotein-2; ID: PF3D7_0501200, also called PFE60 or PfE60/PlasmoDB #: PFE0060w) as a potential surface marker of *P*. *falciparum*-infected RBCs. PIESP2 was not detected in the IP-MS analysis of V5-PfGARP complex using anti-V5 mAb (Table 1). Subsequent comparison of wild-type 3D7 parasites with PfGARP knockout lines by GM7mAb IP-MS identified PIESP2 as one of the major hits (Tables 2 and 3). Our previous studies have shown that GM7mAb recognized a single 48-kDa protein in the wild-type 3D7 parasite lysates by immunoblotting (8). Originally, we hypothesized that the 48-kDa band was presumably a processed form of PfGARP since GM7mAb did not recognize the predicted ∼100 kDa endogenous PfGARP by immunoblotting under these conditions (8, 27). To further investigate the origin of 48 kDa band detected by GM7mAb, we directly sequenced the gel band corresponding to the 48 kDa band by mass spectrometry and found multiple peptides derived from PIESP2. No PfGARP peptides were detected by mass spectrometry of the 48 kDa band. These observations are consistent with published evidence that PIESP2 migrates as a 48 kDa band by SDS-PAGE (11, 12, 13, 14, 15). Together, these findings suggested that GM7mAb can detect both endogenous PfGARP and PIESP2 by IP-MS, but only PIESP2 is detectable as a 48 kDa band by immunoblotting analysis of wild-type *P*. *falciparum* iRBCs.

### Biochemical validation of PIESP2 as the target of GM7mAb

**Figure 4:**
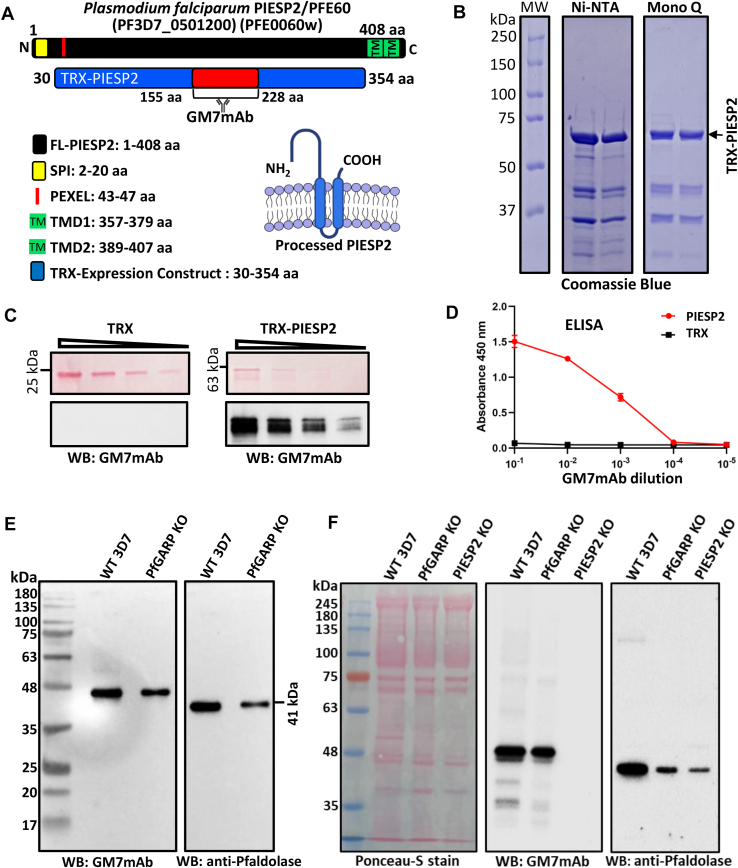
**Identification and validation of PIESP2 by GM7mAb.***A*, the codon-optimized PIESP2 construct was synthesized lacking two predicted transmembrane domains and a signal peptide. Nomenclature of defined domains, expression construct, and putative topology are shown. *B* TRX-PIESP2 protein containing a His-tag, located after TRX protein, was used for affinity purification and fusion protein was purified by Mono Q FPLC. *C*, Ponceau S-stained image of dose-dependent TRX and TRX-PIESP2, followed by immunoblotting with GM7mAb are shown. *D*, ELISA-based detection of TRX and TRX-PIESP2 by serial dilution of GM7mAb. *E*, immunoblotting with GM7mAb using lysates from wild-type 3D7 parasites and PfGARP knockout lines generated at NIH and HMS (only the NIH knockout lane is shown in the *left panel*). An anti-Pfaldolase antibody was used as a loading control (*right panel*). *F*, immunoblot analysis of PfGARP knockout and PIESP2 knockout parasite lysates probed with GM7mAb. The *left panel* shows the corresponding Ponceau S-stained blot for the immunoblots presented in the *right panels*. The 48 kDa band was detected in the PfGARP knockout lysate but was absent in the PIESP2 knockout lysate.

To confirm the identity of PIESP2 as the target of GM7mAb, a full-length construct of *P*. *falciparum* PIESP2 was codon-optimized, and its predicted extracellular domain (amino acids 30–354 (Fig. 4*A*) was expressed in bacteria and purified (Fig. 4*B*). Using immunoblotting, the recombinant TRX-PIESP2 protein was detected by GM7mAb in a dose-dependent manner (Fig. 4*C*) and by ELISA (Fig. 4*D*). Additional evidence for the recognition of PIESP2 by GM7mAb was obtained by immunoblotting analysis of two independent PfGARP knockout lines. The GM7mAb detected the 48 kDa band in PfGARP knockout parasite lines (Fig. 4*E*). To rule out the possibility that GM7mAb can also detect endogenous PfGARP in the wild-type parasite lysate under non-reducing conditions, immunoblotting was performed under both reducing and non-reducing conditions. No ∼100 kDa PfGARP was detected by GM7mAb under either reducing or non-reducing conditions (Fig. S2). These findings indicate that GM7mAb detects recombinant PfGARP by immunoblotting, whereas endogenous ∼100 kDa PfGARP is not detectable under the same conditions. In contrast, GM7mAb can detect both endogenous PfGARP and PIESP2 by IP-MS analysis (Tables 2 and 3). To validate the specificity of GM7mAb for PIESP2, a PIESP2 knockout parasite line was used that was generously provided by Dr Cowman (WEHI). Immunoblotting confirmed the absence of 48 kDa band in the PIESP2 knockout line by GM7mAb (Fig. 4*F*), consistent with the IP-mass spectrometry data (Tables 2 and 3).

**Figure 5:**
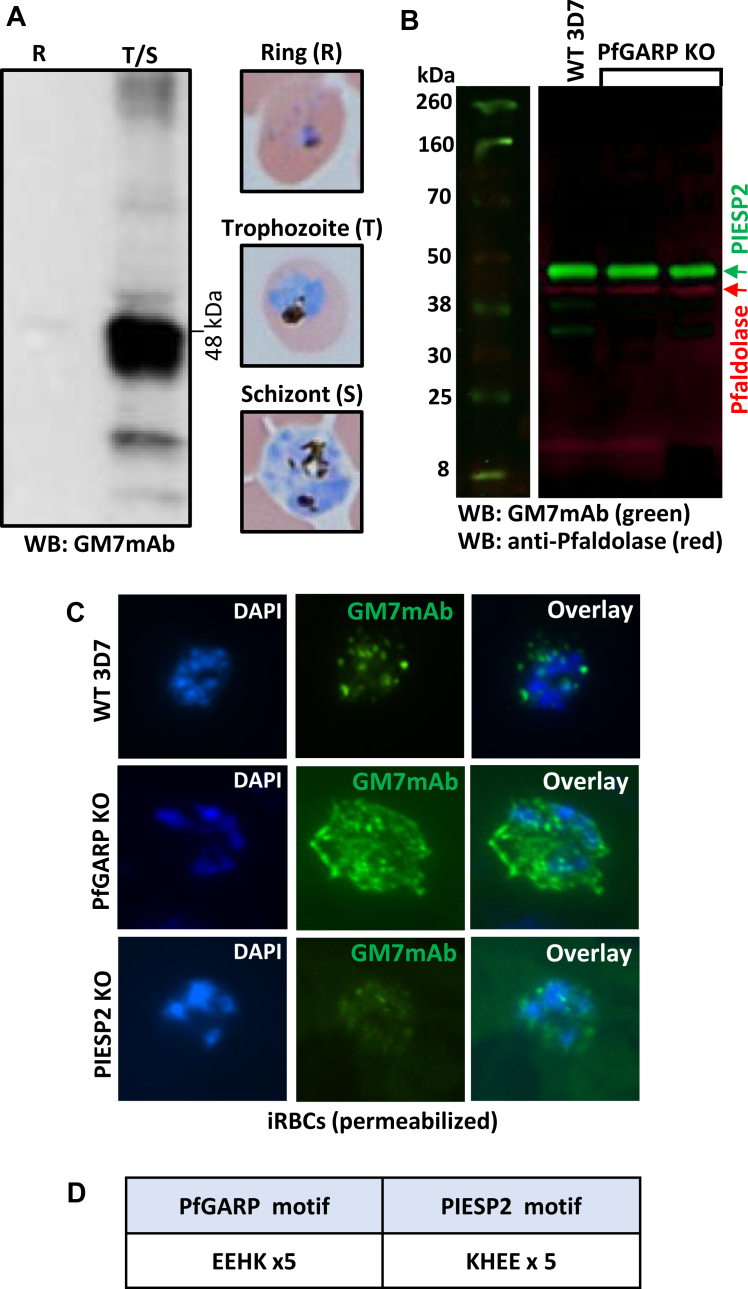
**Localization of PIESP2 in *P*. *falciparum* infected erythrocytes.***A*, using GM7mAb, ring stage and mixed trophozoite/schizont stage infected RBCs were tested by immunoblotting for temporal expression of PIESP2. *B*, multiplex immunoblotting of RIPA-lysed wild-type 3D7 and PfGARP KO (NIH and HMS) parasite lysates. Blots were probed with GM7mAb and rabbit anti-Pfaldolase. Signals were detected by Odyssey CLx using conjugated anti-mouse and anti-rabbit fluorescent secondary antibodies. *C*, IFA of GM7mAb reactivity was performed in wild-type 3D7, PfGARP knockout (HMS), and PIESP2 knockout erythrocytes. Both iRBCs and uninfected RBC s (data not shown) were fixed and permeabilized with cold methanol (−20 ^o^C for 45 min), labeled with GM7mAb and visualized with Alexa Fluor 488-conjugated anti-mouse IgG. Parasite nuclei were labeled with DAPI. *D*, five reverse motifs found in PfGARP-M1 and PIESP2 are shown.

The 48 kDa PIESP2 was detected by GM7mAb in the magnetically purified trophozoites and schizonts but not in ring stage parasites (Fig. 5*A*). Using the PfGARP knockout line, immunoblotting by GM7mAb confirmed the presence PIESP2 in both wild-type 3D7 and PfGARP knockout line (Fig. 5*B*). Immunofluorescence analysis of fixed and permeabilized PfGARP and PIESP2 knockout parasite lines by GM7mAb showed a similar punctate signal as observed in the wild-type 3D7 cells (Fig. 5*C*). These observations are consistent with our finding that GM7mAb recognizes both native PfGARP and PIESP2 by immunofluorescence as well as immunoprecipitation methods but only detects native PIESP2 by immunoblotting assay. Previously, we mapped the epitope recognized by GM7mAb within the PfGARP-M1 segment using a series of synthetic peptides narrowing down the immunoreactive sequence within EEHKE-GEHKE repeats (8). The primary sequence of PIESP2 revealed that it also contained multiple repeats with the KHEE sequence signature. To contextualize these findings, the PIESP2 sequence contained five reverse repeats “KHEE” that are present as “EEHK” in the primary structure of the PfGARP-M1 segment (Fig. 5*D*). These observations suggest that GM7mAb recognize structurally mirrored reverse epitopes present in both the PfGARP and PIESP2 antigens.

### Localization of PIESP2 on the surface of *P*. *falciparum* infected erythrocytes

**Figure 6:**
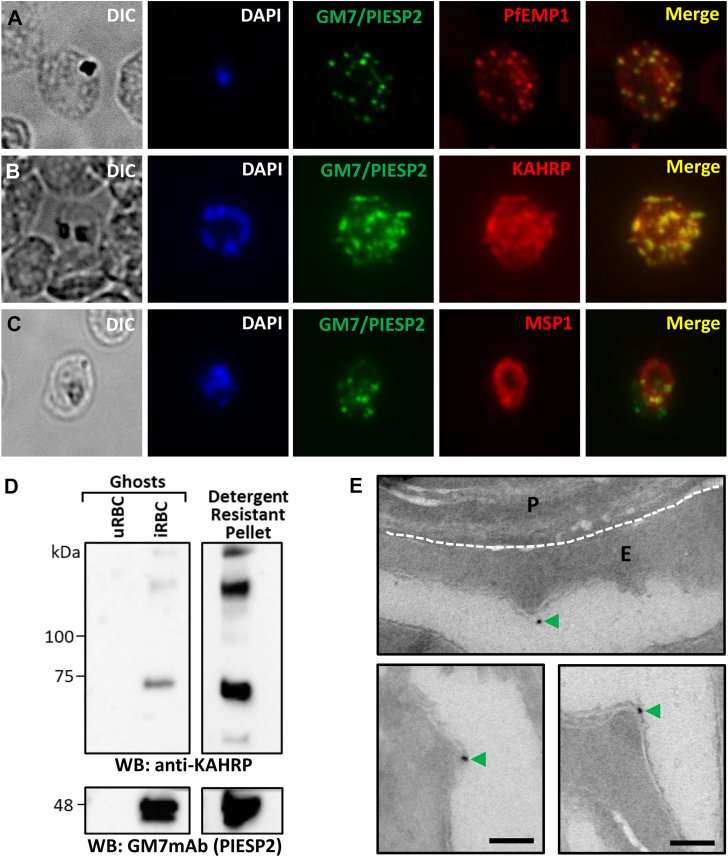
**PIESP2 is a component of knobs.***A–C*, immunofluorescence (IFA) colocalization of PIESP2 using GM7mAb with two known knob markers (PfEMP1 and KAHRP) in wild-type 3D7 iRBCs. MSP1 was used as a negative control. Parasite nuclei were stained with DAPI. iRBCs were fixed and permeabilized with cold methanol (−20 ^o^C for 45 min). *D*, immunoblotting with anti-KAHRP rabbit pAb and anti-PIESP2 (GM7mAb) using uRBC ghosts, iRBC ghosts, and biochemically enriched knobs. PIESP2 is detected in iRBCs and detergent-resistant knobs. *E*, immunogold transmission electron microscopy of wild-type 3D7 parasite infected erythrocytes with GM7mAb. Signal was detected using colloidal gold-labeled secondary antibody. *Green* arrowheads denote PIESP2 signal at the tip of knobs. Letter P refers to intracellular parasite and E shows erythrocyte cytoplasm. Scale bars 100 nm.

Immunofluorescence analysis (IFA) revealed a punctate pattern of PIESP2 expression during the trophozoite and schizont stages of *P*. *falciparum* infection (Fig. 6). We hypothesized that these puncta may correspond to cytoadherent knobs, and co-stained with two established knob markers, PfEMP1 and KAHRP. The data show strong colocalization between PIESP2, PfEMP1, and KAHRP, while the negative control MSP1 exhibited essentially no overlap (Fig. 6, *A*–*C*). To further investigate PIESP2 localization to knobs, the knob fraction was biochemically purified from schizont-infected RBCs as described previously (29, 30). The presence of knobs was confirmed by immunoblotting using a known knob marker KAHRP (Fig. 6*D*). The presence of 48 kDa PIESP2 in the knob-enriched fraction was confirmed by immunoblotting using GM7mAb (Fig. 6*D*). In addition, immunogold transmission electron microscopy (TEM) was used to demonstrate the localization of PIESP2 by GM7mAb at the surface of knobs in iRBCs (Fig. 6*E*). We recognize that additional studies will be needed to evaluate the immunogold TEM localization of PIESP2 using GM7mAb in parasites lacking both PfGARP and PIESP2 generated through targeted gene disruption. These investigations should be complemented by analyses of GM7mAb-mediated PIESP2 localization on knobs in both laboratory-adapted strains and wild-type field isolates of *P*. *falciparum*.

**Figure 7:**
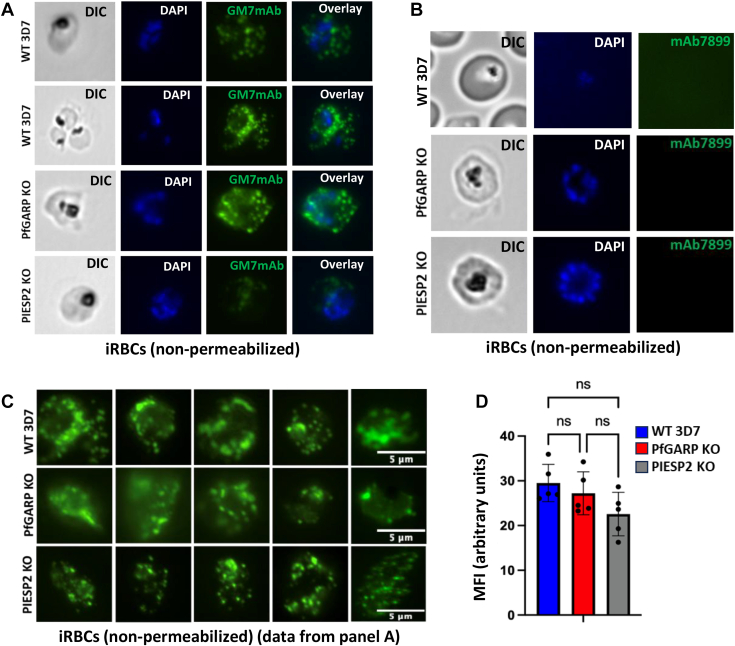
**Localization of PfGARP and PIESP2 on the surface of iRBCs.***A*, differential reactivity of GM7mAb was measured in non-permeabilized wild-type 3D7, PfGARP knockout, and PIESP2 knockout erythrocytes by immunofluorescence assays (IFA). *B*, mAb7899 served as a negative control. *C* and *D*, a montage of representative IFA images for each genotype is shown in *panel C*. The image displayed in the second WT 3D7/GM7 mAb panel of Figure 7*A* is identical to the image shown in the first WT 3D7 panel of Figure 7*C*. To quantify the GM7mAb signal observed in panel A, a larger set of images was analyzed. For quantification, individual infected erythrocytes were manually outlined in ImageJ based on visible fluorescence signal. For each cell, mean fluorescence intensity (MFI) was calculated by subtracting the corresponding background signal. Five representative cells per group were analyzed. Data are presented as mean ± SD. Additional details of IFA quantification are described in the Methods section.

There is prior evidence for a functional role of PIESP2 in intracellular trafficking mediated by Maurer’s clefts as well as its localization on the surface of iRBCs (11, 12, 13, 14, 15, 16, 17, 18). Our identification of PIESP2 by GM7mAb enabled us to investigate its surface localization in intact iRBCs. The punctate signals detected by GM7mAb were observed in the permeabilized iRBCs (Fig. 5*D*). To rule out the possibility that permeabilization may have enabled access of GM7mAb to the intraerythrocytic parasite compartments, including Maurer’s clefts, we performed IFA of iRBCs that were not subject to permeabilization (Fig. 7, *A*–*C*). The punctate signal with GM7mAb was observed in the wild-type 3D7, PfGARP, and PIESP2 knockout lines (Fig. 7, *A* and *C*). The wild-type and PfGARP knockout parasites exhibited both diffuse and punctate surface labeling of infected erythrocytes, whereas the PIESP2 knockout parasites showed a reduction in the diffuse surface signal. This observation suggests that GARP and PIESP2 may exhibit distinct distributions on the host cell surface, consistent with the unbiased mass spectrometry data presented in Table 1, Table 2, Table 3.

Quantification of the mean fluorescence intensities of the stained cells showed that GM7mAb stained signal of PIESP2 KO cells is weaker than wild-type 3D7 line, but not statistically significant (Fig. 7*D*). Of note, the IFA suggests that the puncta detected by GM7mAb in *PfGARP* knockout cells are relatively prominent, possibly reflecting either protein self-association or higher affinity of PIESP2 for GM7mAb. No signal was detected by mAb7899 under identical conditions (Fig. 7*B*). This observation is consistent with our recent demonstration that mAb7899 does not detect PfGARP signal in the fixed and permeabilized *P*. *falciparum* 3D7 iRBCs (27). Future studies will reconcile the technical factors that prevented the detection of intense ring-like membrane staining of PfGARP by mAb7899 in the wild-type *P*. *falciparum* 3D7 iRBCs as reported in an earlier study (10).

**Figure 8:**
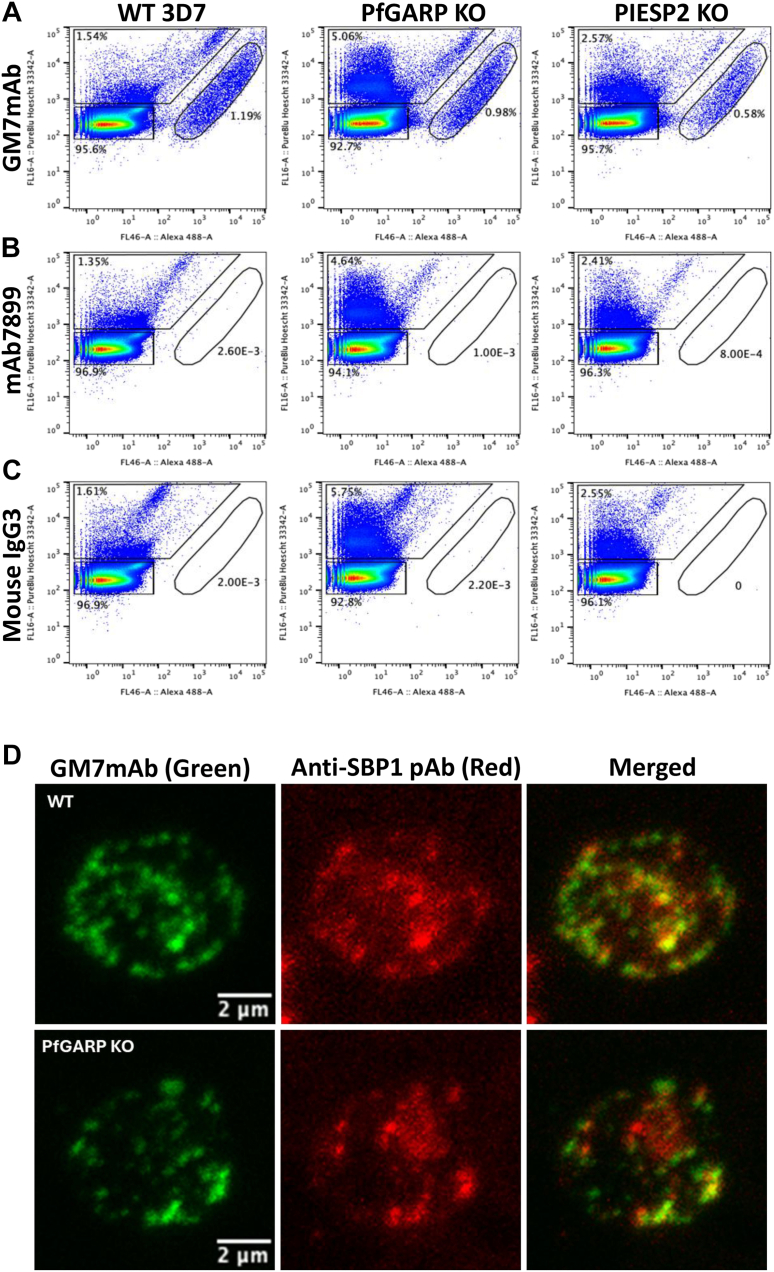
**Quantification of GM7mAb reactivity by flow cytometry. Distinct localization of PIESP2 and skeleton binding protein 1.***A*, flow cytometry was performed on live and unfixed iRBCs from wild-type 3D7, PfGARP knockout, and PIESP2 knockout parasites by GM7mAb to measure surface reactivity. GM7mAb detected robust signal in wild-type 3D7 and PfGARP knockout iRBCs and relatively reduced signal in PIESP2 knockout iRBCs. The numerical values at the right of each panel represent mature parasites. Details of data analysis by FlowJo Software and gating strategies are described in the Methods section. *B* and *C*, mAb7899 and mouse IgG3 isotype were used as negative controls. *D*, immunofluorescence assays (IFA) were used to compare the localization of GM7mAb reactivity (PIESP2) with rabbit anti-SBP1 pAb antibody in wild-type 3D7 and PfGARP knockout iRBCs by confocal microscopy. For confocal microscopy using anti-SBP1 pAb, iRBCs were air-dried and fixed at room temperature with 100% acetone for two minutes (details in Experimental procedure). Representative images of wild-type 3D7 and PfGARP KO parasites subject to co-staining are shown in Figure S4. The IFA signals were detected by Alexa Fluor 488 anti-mouse secondary antibody and Alexa Fluor 568 anti-rabbit secondary antibody, respectively. Images were acquired on a Zeiss LSM 880 confocal microscope equipped with a 63 × /1.4 NA Plan-Apochromat oil-immersion objective. Scale bar: 2 μm.

The surface localization of PIESP2 and PfGARP in iRBCs was also evaluated by flow cytometry (Fig. 8*A*). Intact untreated wild-type *P*. *falciparum* 3D7, PfGARP and PIESP2 knockout lines were incubated with GM7mAb, and surface reactivity of the antibody was quantified. Flow cytometry detected surface expression of both PIESP2 and PfGARP, consistent with the recognition of reverse epitopes by GM7mAb. No signal by flow cytometry was detected either with mAb7899 (Fig. 8*B*) or IgG3 (Fig. 8*C*) under identical conditions. Moreover, unfixed human erythrocytes were assessed for reactivity with GM7mAb. No positive signal was observed on uninfected cells, demonstrating that GM7mAb does not recognize negatively charged structures on the surface of normal RBCs (Fig. S3).

Finally, we used a polyclonal antibody raised against parasite-derived skeleton-binding protein 1 (SBP1) (31) to compare its localization in Maurer’s clefts with PIESP2. Since GM7mAb recognizes reverse epitopes in PfGARP and PIESP2, we performed IFA in both wild-type 3D7 and PfGARP knockout lines by confocal microscopy (Fig. 8*D*). A montage of images from the same experiment is also shown (Fig. S4). SBP1 exhibited a predominantly punctate distribution throughout the cytoplasm in wild-type 3D7 iRBCs (Red signal in Fig. 8*D* (Fig. S4). In contrast, SBP1 staining appeared more confined to the parasitophorous vacuole membrane (PVM) in the PfGARP knockout cells (Fig. 8*D*). The GM7mAb signal was largely adjacent to SBP1, consistent with the model that PIESP2 localizes to both Maurer’s clefts and surface knobs in iRBCs (Green signal in Fig. 8*D* and Fig. S4). The differential localization of SBP1 between wild-type 3D7 and PfGARP knockout parasites is more evident in the cell montage images presented in Figure S4, raising the possibility that PfGARP plays a functional role in SBP1 trafficking or Maurer’s cleft formation. Collectively, these findings support our model that a subset of PIESP2 localizes to the surface of mature parasite-infected erythrocytes.

### Detection of PIESP2 antibodies in human plasma by ELISA

**Figure 9:**
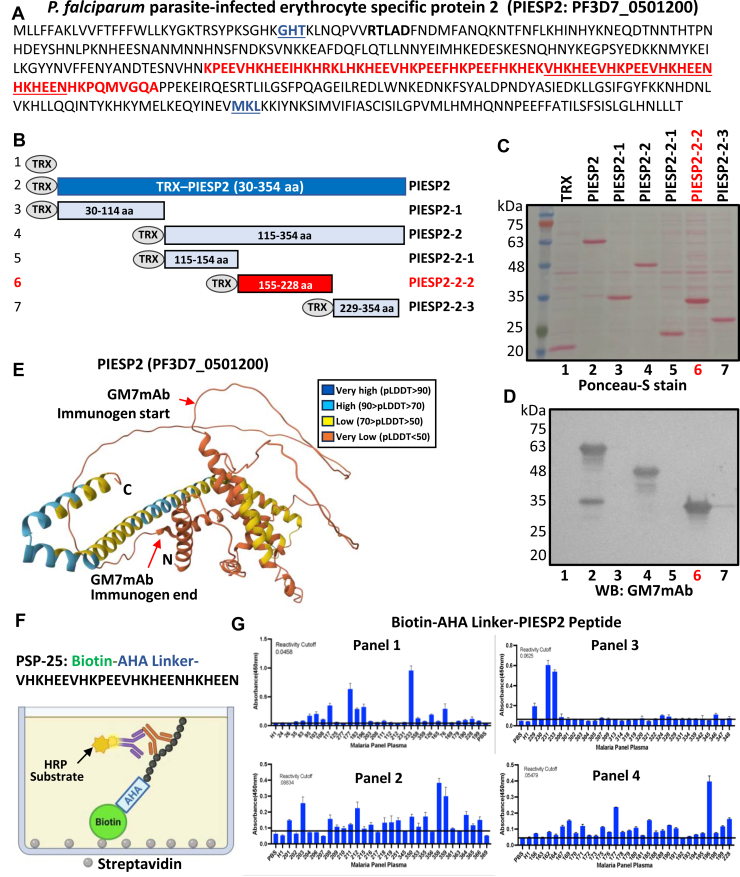
**Detection of antibodies in human malaria plasma samples by PIESP2 immunoassay.***A*, *blue color-coded* three amino acids denote the boundaries of TRX-PIESP2 construct in the full-length PIESP2 (PF3D7_0501200) sequence. *Red color-coded* amino acids denote the PIESP2 segment recognized by GM7mAb. Bold RTLAD sequence denotes the location of PEXEL motif. *B*, mapping of GM7mAb recognition segment within PIESP2-2-2 construct (amin acids 155–228). *C*, Ponceau-S staining of PIESP2 constructs shown in *panel B*. *D*, immunoblotting of PIESP2 constructs (panel C) by GM7mAb. *E*, alphaFold predicted location of PIESP2-2-2 segment recognized by GM7mAb. *F*, development of an ELISA for the detection of antibodies against PIESP2. Guided by the presence of reverse motifs in PIESP2, a 25-amino acid peptide (PSP-25) contained within PIESP2-2-2 was synthesized and conjugated to biotin *via* a flexible linker. The specificity of the peptide was validated by ELISA using GM7mAb and TRX-PIESP2 fusion protein (Fig. S5). *G*, ELISA screens of human plasma samples from a malaria-endemic region in Mali, Africa. Negative controls of PBS and uninfected human serum (H1) were included. The reactivity cutoff value is shown in each ELISA panel. Several plasma samples including 169, 179, 190, 199, 223, 228 were reconfirmed by multiple ELISA screens. Additional details are described in the Experimental procedure section.

The surface exposure of PIESP2 by IFA of non-permeabilized PfGARP knockout iRBCs, immunogold localization of GM7mAb reactivity on knobs, and robust PIESP2 signal detection by flow cytometry in intact PfGARP knockout iRBCs, motivated us to evaluate for the presence of PIESP2 antibodies in a malaria-endemic region in Africa. Previously, we have screened over 300 plasma samples from Kambila, Mali, obtained from the NIH-NIAID, to identify potential biomarkers of malaria infection in the endemic areas (8, 32). Kambila is a rural village in Mali where *P*. *falciparum* transmission is seasonal and severe (32). Plasma samples represented both male and female donors in addition to young children exposed to malaria (32). Using recombinant TRX-PIESP2 extracellular domain (Fig. 9*A*, amino acids from Gly 30 to Leu 354), an ELISA-based screen of 84 plasma samples showed a positive identification rate of 74% with a cutoff value of 0.54 (Fig. S5, panels A, B). While encouraging, the high background signals observed in ELISA screens using immobilized recombinant TRX-PIESP2 antigen prompted us to develop a more quantitative, peptide-based ELISA that can be readily reproduced for the detection of PIESP2 antibodies.

A series of conjoint and overlapping TRX-PIESP2 constructs were expressed in bacteria (Fig. 9*B*) and tested by immunoblotting using GM7mAb (Fig. 9, *C* and *D*). Only the TRX-PIESP2-2-2 construct from amino acids 155 to 228 was recognized by the GM7mAb (Fig. 9*B*, red color). Interestingly, the entire PIESP2-2-2 segment containing the immune epitope was located within a flexible and non-structured region of PIESP2 as predicted by AlphaFold (Fig. 9*E*). Importantly, the PIESP2-2-2 segment contained the reverse KHEE epitopes recognized by GM7mAb. Guided by this information and *in silico* peptide scanning, a 25-amino acid peptide from the PIESP2 antigen was designed and chemically synthesized. The synthetic peptide termed PSP-25 contained an N-terminal flexible linker AHA (aminohexanoic acid) coupled to biotin (Fig. 9*F*). The reactivity and specificity of PSP-25 peptide against GM7mAb was validated using both positive and negative peptide controls derived from PfGARP (8) (Fig. S5*C*). Biotinylated PSP-25 peptide was immobilized to Streptavidin-coated ELISA plates to develop a quantitative ELISA for measuring antibodies against PIESP2 in the human plasma samples (Fig. 9*F*). As described above, multiple screens of 100 plasma samples originating from the malaria endemic region in Mali identified several samples that were highly reactive to PSP-25 peptide (Fig. 9*G*). These results demonstrated the development of a quantitative ELISA with relatively low background signal that will allow detection of antibodies against PIESP2 in the malaria endemic regions. Future adaptation of the 25-amino acid PSP-25 synthetic peptide and GM7mAb is expected to facilitate the development of simple, rapid, and cost-effective diagnostic platforms, including lateral flow assays, for detecting PIESP2 antibodies during acute malaria infection.

### Potential role of PIESP2 and PfGARP in cytoadhesion

**Figure 10:**
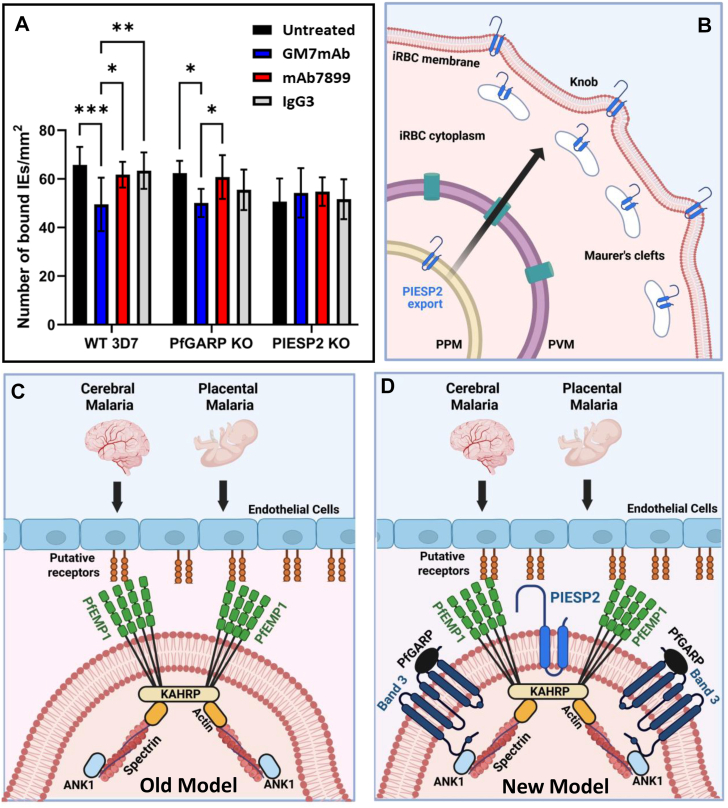
**Cytoadhesion and schematic representation of knob topology.***A*, wild-type 3D7, PfGARP knockout, and PIESP2 knockout parasites were evaluated for static binding to human cerebral microvascular endothelial cells (HBEC-5i-CRL-3245 line. ATCC). Bound iRBCs were fixed, stained with Wright-Giemsa, and quantified by microscopy. Effect of GM7mAb, mAb7899, and mouse IgG3 isotype was measured as outlined in the Experimental procedure section. Each parasite line was evaluated in a minimum of three independent experiments. *B*, schematic representation of PIESP2 trafficking to knobs *via* Maurer’s clefts in infected erythrocytes. *C* and *D*, schematic representation of parasite-derived adhesion proteins including PfEMP1, PIESP2, and PfGARP anchored on knobs as putative ligands for host endothelial receptors.

There is evidence for the surface localization of PfGARP in iRBCs (8, 10, 33). Our previous findings suggested a functional role of PfGARP in erythrocyte rosetting (8), but its direct role in RBC adhesion to endothelial cells is lacking. Similarly, there is substantial evidence supporting a functional role for PIESP2 in cellular adhesion (11, 13, 15, 16, 17, 18). A recent study implicated PIESP2 as a virulence factor in cerebral malaria (15). With the characterization of two distinct monoclonal antibodies generated against non-native PfGARP (8, 10) combined with PfGARP and PIESP2 knockout lines, we evaluated the inhibitory effects of GM7mAb and mAb7899 on wild-type 3D7 and knockout parasite lines using a static cell adhesion assay. We used the human HBEC-5i cell line for *in vitro* parasite adhesion assay, which is commonly used to mimic a phenotype resembling brain microvascular endothelial cells (34, 35, 36). Nonetheless, the laboratory-adapted isolates of *P*. *falciparum* often bind poorly to these and other endothelial cell lines (37, 38). We evaluated wild-type 3D7 as well as both knockout parasite lines for binding to HBEC-5i cells using a quantitative peroxidase-based assay (39). Our initial screens showed a modest but significant inhibition of adhesion of laboratory-adapted *P*. *falciparum* 3D7 and PfGARP knockout iRBCs to HBEC-5i cells by GM7mAb but not mAb7899 (Fig. 10*A*). In contrast, no inhibitory effect of GM7mAb and mAb7899 was observed on the adhesion of PIESP2 knockout iRBCs to HBEC-5i cells (Fig. 10*A*). The IgG3 served as a negative control. Although these initial screens suggest a potential role of PfGARP and PIESP2 in cellular adhesion, a more rigorous approach will require repeated selection of defined parasite lines on human endothelial cells as described by Rowe and colleagues (37). Quantification of parasite-mediated cellular adhesion using field isolates obtained from malaria-infected patients will be essential to clarify the inhibitory effect of GM7mAb under shear stress on the physiologically relevant binding of infected erythrocytes (iRBCs) to endothelial receptors and chondroitin sulfate A relevant to cerebral and placental associated malaria, which are implicated in cerebral and placental malaria.

## Discussion

A unique characteristic of *P*. *falciparum* is the creation of adhesive knobs on the surface of infected red blood cells (iRBCs or erythrocytes) that enables parasite attachment to microvasculature and deep tissue sequestration during infection. While knobs are both necessary and sufficient for adherence of iRBCs, the ensuing acute malaria pathology is multifactorial, involving inflammation, ischemia, and thrombotic lesions. The current paradigm invokes that the intraerythrocytic *P*. *falciparum* synthesizes a key virulence protein termed PfEMP1 (*P*. *falciparum* erythrocyte membrane protein 1) that is anchored on the surface of knobs in iRBCs (5, 6, 7, 40). Although PfEMP1 was discovered more than 30 years ago, no therapeutic approach has yet succeeded in clinically suppressing the adhesion of iRBCs to endothelial cells *in vivo*. This limitation is mainly attributed to the highly polymorphic nature of PfEMP1 encoded by ∼60 *var* genes (41). Here, we provide evidence for the localization of PIESP2 (Parasite-Infected Erythrocyte Surface Protein 2), a putative transmembrane protein encoded by a single-copy gene in the *P*. *falciparum* genome, on the surface of infected red blood cells (iRBCs), suggesting its potential role as a novel component of the knob adhesion complex (Fig. 10, *B*–*D*).

The serendipitous identification of PIESP2 as a surface protein originated from our discovery of PfGARP’s role in rosette formation (8). We identified two distinct repetitive regions in PfGARP termed PfGARP-M1 and PfGARP-M2. The PfGARP-M1 region emerged as highly immunogenic and exhibited sequence variation among parasite isolates. In contrast, the conserved PfGARP-M2 region directly interacted with intact RBCs (8). Subsequent studies showed that PfGARP binds to a specific region of RBC band 3 (anion exchanger-1/SLC4A1) that has been characterized as a neoantigen (pfalhesin) mediating parasite attachment to thrombospondin and CD36 (8, 23, 25, 26). Since band 3 is the most abundant multi-transmembrane oligomeric protein in erythrocytes (42, 43), we surmised that PfGARP may form oligomers, forming a macromolecular complex with clustered band 3. Biochemical evidence presented in this study supports the model that PfGARP self-assembles into oligomers, and its binding to intact RBCs is differentially modulated by Zinc and DIDS (Fig. 2, *G* and *H*). Since the intramembrane particles (IMPs) composed of band 3 and glycophorin A (GPA) decorate the entire surface of erythrocytes, including knobs (red arrows, Fig. 3*F*), our findings support a model for the presence of oligomeric PfGARP-Band 3 complex at the periphery of iRBCs including knobs.

A puzzling aspect of PfGARP biology is the lack of antibodies including GM7mAb that can detect full-length endogenous PfGARP by immunoblotting under both reducing and non-reducing conditions (Fig. S2). The original identification of PfGARP by Kemp and colleagues (44) reported that they could not detect endogenous PfGARP by immunoblotting using affinity purified antibodies harvested from patients sera (44). A subsequent study used anti-GFP antibodies to localize PfGARP to the periphery of transfected iRBCs (33). Later another study identified a ∼100 kDa band of endogenous PfGARP in iRBCs by immunoblotting using a monoclonal antibody called mAb7899 (10). However, our multiple attempts to detect full-length endogenous PfGARP in iRBCs by immunoblotting methods using the same mAb7899 have not been successful under the identical conditions reported in the previous study (27). Consistent with this observation, our flow cytometry data showed no detectable surface-exposed PfGARP on unfixed live iRBCs when probed with mAb7899 (Fig. 8*B*). It remains possible that the conformational epitope(s) recognized by mAb7899 within the oligomeric PfGARP-Band 3 complex was masked under the experimental conditions used in this study. The availability of mAb7899 will facilitate future efforts to optimize detection conditions for full-length endogenous PfGARP.

Since our GM7mAb raised against recombinant PfGARP-M also did not detect the full-length ∼100 kDa band of endogenous PfGARP by immunoblotting (8, 27), we utilized the V5-PfGARP transfected iRBCs (10, 27) to investigate the recognition of V5-tagged PfGARP by anti-V5mAb and GM7mAb (Fig. 3). The technical details of the V5-PfGARP expression system have been reported previously (10). The V5-PfGARP system generated multiple translation products, and a stable V-PfGARP band migrating as ∼165 kDa likely reflects the highly charged nature of PfGARP (Fig. 3). Both anti-V5mAb and GM7mAb efficiently removed soluble V5-PfGARP from the parasite lysates (Fig. 3). The magnetic beads containing the immunoprecipitated material were analyzed by unbiased mass spectrometry (Tables 1 and 2). As expected, V5-PfGARP and host band 3 were identified as major parasite and host components of the V5-PfGARP complex using anti-V5 mAb (Table 1). Host spectrin and ankyrin-1 were identified, consistent with their known interactions with the band 3 complex (42, 45). Two exported malaria proteins, MESA and glycophorin-binding protein (GBP-130), were also identified in the beads coated with anti-V5 mAb (Table 1). A specific motif in MESA is known to bind to ankyrin-1, thus suggesting the possibility that MESA may also be associated with the PfGARP-Band 3-Ankyrin complex (45). Similarly, glycophorin A tightly associated with band 3 form a complex with GBP-130, and the GBP130-GPA-Band 3 complex may regulate the rigidity and stability of iRBCs consistent with the increased membrane rigidity observed in the GBP-130 knockout parasite line (12). The schematic of the V5-PfGARP interactome is summarized in Figure 3*G*. The availability of two independent PfGARP knockout lines outlined in this study will begin to clarify the biochemical basis of PfGARP complex assembly in iRBCs.

To compare the composition of V5-PfGARP interactome (Table 1), the V5-PfGARP lysate was immunoprecipitated with GM7mAb and analyzed by unbiased mass spectrometry. The rationale of this approach was to determine whether GM7mAb can recognize the natively folded PfGARP interactome under the same conditions. The endogenous PfGARP was pulled down by GM7mAb as a major protein from V5-PfGARP lysate (Table 2). While comparing the two mass spectrometry screens, an unexpected finding was the presence of PIESP2 in the beads coated with GM7mAb (Table 2) but not in the anti-V5 mAb screen (Table 1). To further investigate this intriguing observation, we used GM7mAb to evaluate the composition of wild-type 3D7 line and PfGARP knockout parasite interactomes by IP-mass spectrometry (Table 3). The presence of PIESP2 peptides detected in the PfGARP knockout lysate (Table 3) indicated that GM7mAb is endowed with dual specificity for endogenous PfGARP and PIESP2.

The punctate staining pattern observed with GM7mAb in wild-type, PfGARP-knockout, and PIESP2 knockout parasite-infected erythrocytes (Figure 5, Figure 6, Figure 7) is consistent with previous immunogold localization studies demonstrating GM7mAb reactivity associated with knob structures. Since our data indicate that GM7mAb recognizes both GARP and PIESP2, we would not expect complete loss of signal in parasite lines lacking either antigen individually. Consistent with this expectation, immunofluorescence analysis of fixed and permeabilized PfGARP and PIESP2 knockout parasites using GM7mAb revealed a punctate staining pattern similar to that observed in wild-type 3D7 parasites (Figs. 5 and 7). Since immunofluorescence imaging is susceptible to fixation-dependent artifacts and because staining patterns can vary among individual infected cells due to differences in parasite developmental stage and epigenetic heterogeneity within clonal cultures, we did not perform a detailed quantitative analysis of these staining differences. Instead, we included five representative images for each genotype to illustrate the observed patterns (Fig. 7*C*). Definitive localization of PfGARP and PIESP2 within knob structures will require the generation of parasites lacking both genes, along with the use of highly specific, validated monoclonal antibodies for PfGARP and PIESP2 in immunofluorescence and electron microscopy studies.

Several previous studies have shown that PIESP2 migrates as a 49-kDa protein by immunoblotting analysis of parasite lysates (11, 14, 15). Originally, we developed GM7mAb against recombinant PfGARP-M protein and detected a 48-kDa protein by immunoblotting of *P*. *falciparum* lysates (8). In this study, we provide comprehensive evidence to demonstrate that PIESP2 is the sole 48-kDa antigen detected by GM7mAb in the *P*. *falciparum* lysates. GM7mAb can recognize both endogenous PIESP2 and PfGARP using immunoprecipitation, immunofluorescence and flow cytometry assays. The unique dual specificity of GM7mAb originated from the reverse epitopes located in the PfGARP-M1 and extracellular domain of PIESP2. The presence of reverse motifs in PIESP2 was confirmed by the domain mapping studies by GM7mAb. A synthetic peptide containing the reverse epitope of PIESP2 was used to develop a quantitative ELISA that successfully quantified immune response against PIESP2 in plasma samples collected from patients in a malaria-endemic area in Africa (Fig. 9).

It may be important to discuss the nuances of the existing PIESP2 literature in relation to the findings presented in this study. A previous study failed to detect PIESP2 in a purified fraction of knobs by proteomics analysis (29). The same study also failed to detect PfEMP1 in knobs consistent with known technical challenges in identifying certain transmembrane proteins by proteomics analysis of gel bands excised from SDS-PAGE (29). The presence of PfEMP1 as a major component of knobs is now well established (46, 47, 48). There is ample evidence supporting the location of PIESP2 on the surface of iRBCs (15, 17, 18, 49, 50) as well as a resident protein on Maurer’s clefts (11, 14, 16). A recent study, published as a preprint online, investigated the role of MSRP6 in Maurer’s clefts and concluded that loss of Maurer’s clefts anchoring is neither needed for PfEMP1 transport nor cytoadherence (19). This study implicated MSRP6 complex with PIESP2 and other effectors in a stage-specific anchoring of Maurer’s clefts that is independent of PfEMP1 transport (19). While the precise biological function of Maurer’s clefts remains open to future investigations, our finding that GM7mAb localization is mostly adjacent to SBP1 (Figs. 8*D* and S4) opens the possibility that PIESP2 and other knob proteins, including PfEMP1, could utilize Maurer’s clefts independent transport pathways to reach their destination of host erythrocyte surface. Our study addresses an important gap through the identification of PIESP2 by GM7mAb using immunoblotting, immunofluorescence, immunoprecipitation, mass spectrometry, flow cytometry, and immunogold transmission electron microscopy. Future studies are needed to determine whether this unique monoclonal antibody can serve as a reliable marker for knobs in *P*. *falciparum*-infected human erythrocytes.

A recent study identified PIESP2 as a potential antigen involved in the progression of cerebral malaria (15). This study found a pool of PIESP2 localized to the surface of iRBCs during the late stages of parasite development and mediated iRBCs attachment to vascular endothelium (15). Polyclonal serum generated against PIESP2 demonstrated ∼70% inhibition of iRBCs attachment to brain endothelial cells (15). Moreover, recombinant PIESP2 was shown to bind the extracellular surface of host endothelial cells (15). While our initial static adhesion assays suggest that PfGARP and PIESP2 play functional roles in the adhesion of iRBCs to endothelial cells (Fig. 10*A*), the availability of GM7mAb will facilitate the identification of the specific endothelial receptor(s) for both PIESP2 and PfGARP. An in-depth characterization of the PIESP2-endothelial interface would constitute a major advance in understanding host-pathogen interactions mediated by the PfEMP1 family of adhesion receptors anchored on knobs (Fig. 10, *B*–*D*).

While alternative models such as rodent *Plasmodium berghei* ANKA have been investigated for cerebral malaria (51), both PIESP2 and PfGARP are specific to the Laveranian genus of *Plasmodium*, including *P*. *falciparum* and *Plasmodium knowlesi*, and therefore more suitable for *in vivo* testing in non-human primate models. The exact etiology of cerebral malaria remains complex. Future development of inhibitory antibodies targeting PIESP2, PfGARP, and PfEMP1 may suppress the inflammatory response to microvascular endothelial cells in patients with severe malaria (15). A combination of targeting proinflammatory and cytoadhesion mechanisms may identify PIESP2 as an attractive therapeutic target against cerebral and pregnancy-associated malaria.

## Experimental procedures

### Malaria parasite culture

*P*. *falciparum* wild-type 3D7 (Malaria Research and Reference Reagent Resource Center, Manassas, VA), PfGARP knockout and V5-tagged PfGARP 3D7 (Jeffrey D. Dvorin, Harvard Medical School; HMS), PfGARP knockout (Dr Sanjay Desai, National Institute of Health; NIH), and PIESP2 knockout (12, 13) (Sr. Alan F. Cowman at the Walter and Eliza Hall Institute of Medical Research) parasites were cultured *in vitro* in complete malaria media (CMM) containing RPMI-1640 with 2 mM L-glutamine (Gibco) supplemented with 0.5% Albumax II, 25 mM HEPES, 50 mg/L hypoxanthine, and 50 mg/L gentamicin in a 37 °C New Brunswick Galaxy 48 R incubator (Eppendorf) maintained with a gas mixture of 5% CO_2_, 3% O_2_, and balanced by N_2_. Anonymized human red blood cells (RBCs), obtained from the blood bank, were washed with RPMI-1640 at least three times, stored at 50% hematocrit in RPMI at 4 to 8 °C, and used within 2 weeks. Blood smears were fixed with 100% methanol for 30 s, air-dried, stained by Wright Giemsa, and parasitemia was quantified by microscopy (60 × magnification, oil immersion). A standard Percoll/Sorbitol synchronization method was used to tightly maintain culture synchrony. Schizonts were enriched by a two-step Percoll (Sigma) density gradient and isolated schizonts were allowed to reinvade RBCs for 6 to 12 h before the resulting ring-stage parasites were subjected to 5% D-sorbitol (Sigma) treatment. To harvest synchronized parasites and culture supernatant, RBCs infected with mature-stage parasites from wild-type 3D7 (WT), PfGARP knockout (NIH and HMS), PIESP2 knockout, and V5-tagged PfGARP 3D7 cultures were purified using a Miltenyi Biotec LS magnetic column (magnetic-activated cell sorting; MACS).

### Blood and IRB

This research uses only de-identified blood cells obtained from the blood bank. *In vitro* studies will utilize blood cells from both male and female adult blood donors. Human blood used for malaria research *in vitro* is considered as “not Human Subjects Research” because human subjects donating blood are not identifiable by the researchers. The Tufts Institutional Review Board has reviewed and approved the protocol (MODCR-07-9750). PI: A. Chishti. “The regulation of membrane-protein interactions in blood cells” for using human blood cells including platelets. The IRB protocol is valid until 08/25/2026 subject to renewal each year.

### Enrichment of knobby malaria-infected erythrocytes using gelatin flotation

Regular selection of parasite cultures to enhance and maintain knob expression was performed as described (52, 53). Trophozoite and schizont mature-stage *P*. *falciparum* infected erythrocytes (IEs) were centrifuged at 800*g* for 4 min and washed once with pre-warmed RPMI-1640. Parasite pellets were resuspended in 4 volumes of pre-warmed, sterile-filtered 2% gelatin (Sigma) in RPMI-1640 with 25 mM HEPES and 2 mM L-glutamine and incubated for 45 min at 37 °C. Supernatant containing the knobby parasites was carefully removed without disturbing the pelleted ring forms, rosettes, and knobless mature stages. Knobby parasites were washed at least three times with warm RPMI-1640 medium and then subcultured. This procedure was repeated each week to further enrich the knobby phenotype. The presence of knobs was confirmed by electron microscopy, immunofluorescence, and immunoblotting with a known knob marker. Wild-type and knockout parasite cultures used for cytoadhesion assays were subject to at least 6 months of knob selection using gelatin flotation.

### Human brain endothelial cells culture

HBEC-5i endothelial cells (from American Type Culture Collection (ATCC; catalogue number CRL-3245) were cultured in complete endothelial medium (Dulbecco's Modified Eagle Medium: nutrient Mixture F-12 (DMEM-F12), supplemented with 10% FBS, 40 μg/ml endothelial cell growth supplement (ECGS), 1% penicillin-streptomycin solution) under 5% CO_2_ at 37 °C in 75 cm^2^ flasks or 6-well tissue culture plates coated with 0.1% gelatin (Millipore), according to manufacturer’s instructions. HBEC-5i were cultured until 80% confluent with daily medium changes and were used between passages 4 and 6. Unless stated otherwise, solutions were prepared using analytical grade reagents and sterilized using a 0.22-μm filter unit.

### Selection of malaria-infected erythrocytes (IEs or iRBCs) binding to HBEC-5i

To mimic cerebral malaria infection *in vivo*, we followed an established assay (37) to select for parasites that bind to HBEC-5i. This method allows for repetitive selection and subsequent culturing of parasite populations expressing surface antigens that bind to HBEC-5i at higher proportions than unselected populations. High parasitemia (at least 10%) cultures of *P*. *falciparum* wild-type 3D7, PfGARP knockout, and PIESP2 knockout lines at the trophozoite stage were pelleted and washed twice with warm, incomplete DMEM medium (Dulbecco's Modified Eagle Medium: nutrient Mixture F-12 (DMEM-F12), supplemented with 1% penicillin-streptomycin solution). Parasites were resuspended in incomplete DMEM medium supplemented with 3% BSA (GoldBio). Separate HBEC-5i coated petri dishes (80–90% confluency) were washed twice with incomplete DMEM medium before respective solutions of parasites were added to each plate and incubated at 37 °C for 90 min. Twice, during incubation, parasites were gently rocked to resuspend the solution (after 30 min and 60 min). Petri dishes were washed five times with incomplete DMEM medium to remove any unbound uRBCs and iRBCs; additional washes were performed, if necessary, after checking under an inverted microscope for any remaining loose cells. Freshly prepared warm CMM was added to each dish in addition to 40 μl of packed uRBCs and incubated overnight at 37 °C. Parasites were harvested the following day with multiple, vigorous RPMI washes and resuspended to the desired hematocrit for continuous culturing until the next selection. Wild-type and knockout parasites used in static adhesion assays were subject to three rounds of selection.

### Molecular cloning and purification of PfGARP protein constructs

*P*. *falciparum* glutamic acid rich protein (PfGARP) encoding amino acids 356 to 552 (PfGARP-L) was amplified by polymerase chain reaction (PCR) and cloned into the pLIC maltose binding protein (MBP) vector as described (54). Recombinant MBP-GARP-L was transformed into *Escherichia coli* BL21 (DE3) competent cells (Novagen) for bacterial expression and purification. Transformants were grown in Luria Broth (LB) supplemented with 100 μg/ml ampicillin, at 37 °C until optical density at 600 nm (OD_600_) of 6.0 was reached. Cultures were induced with 0.1 mM IPTG and incubated for 2 h at 37 °C. Bacteria were centrifuged and resuspended in lysis buffer with 0.2% Triton X-100, phenylmethyl sulfonyl fluoride (PMSF), and lysozyme. Cells were sonicated and clarified at 13,000*g* for one hour at 4 °C. MBP-GARP-L was purified using a nickel-nitrilotriacetic acid (Ni-NTA) matrix on an ÄKTA fast protein liquid chromatography (FPLC) system (GE Healthcare). Eluates were analyzed by SDS-PAGE, and fractions containing the protein of interest were pooled and buffer exchanged into phosphate-buffered saline (PBS) overnight at 4 °C. The thioredoxin-tagged PfGARP-M recombinant constructs were cloned and expressed as described previously (8). PfGARP-M (AA 370–444) was codon-optimized and cloned into pET32b, and the non-overlapping segments of PfGARP-M, PfGARP-M1 (AA 370–416) and PfGARP-M2 (AA 417–444) were cloned into pET32a vectors (GenScript). All PfGARP-M recombinant proteins were expressed and purified as described above.

### Zinc chloride clustering and DIDS binding

Treatment of uninfected RBCs with zinc chloride was performed as described (22). Briefly, equal amounts of uRBCs were washed in HEPES-buffered saline (HBS) and incubated with 10 μm ZnCl_2_ for 15 min at 37 °C. Treated cells were washed three times with PBS and incubated with an equal amount of MBP-GARP-L to a final concentration of 100 μg/ml for 20 min at 37 °C. Ghosts were prepared from treated uRBCs using hypotonic lysis with 5 mM sodium phosphate and 1 mM EDTA, pH 8.0. Samples were mixed with sodium dodecyl sulfate (SDS) sample buffer with reducing agent β-mercaptoethanol (β-ME) and heated to 95 °C for 10 min, and proteins were separated on 12% or 4 to 20% gradient SDS-PAGE gels. Separated proteins were transferred to nitrocellulose membranes, which were blocked with 5% milk in PBST (0.05% Tween-20) for 1 h at room temperature. Membranes were probed with GM7mAb and anti-band 3, diluted 1:50000 and 1:1000 in blocking buffer, respectively, and detected by anti-mouse-horseradish peroxidase (HRP) immunoglobulin G (IgG).

A 1.4 mM stock solution of DIDS (4,4′-diisothiocyanato-2,2′-stilbenedisulphonate) was prepared in PBS. For PfGARP binding to DIDS-treated uRBCs, uRBCs were incubated with 50 or 100 μm DIDS in PBS for one hour at 37 °C. Treated cells were washed three times with PBS. Treated and untreated uRBCs were incubated with MBP-GARP-L at a final concentration of 100 μg/ml for 30 min at 37 °C. Samples were analyzed by immunoblotting, as described above. Membranes were probed with GM7mAb (1:50000) and anti-p55 mAb (1:5000) and detected by anti-mouse IgG-HRP-conjugated antibody. For enhanced chemiluminescent (ECL) detection, membranes were developed with SuperSignal West Pico PLUS chemiluminescent substrate (Thermo Scientific) and visualized on a ChemiDoc XRS + Imaging System (BioRad) using autoexposure settings.

### CRISPR-mediated generation of PfGARP gene knockout in *P*. *falciparum*

A CRISPR-Cas9 transfection strategy was designed to produce *pfgarp* knockout parasites in the 3D7 and Dd2 parental backgrounds, representing sialic acid-independent and -dependent parasites, respectively (55). This strategy, based on homology directed repair, yielded a 118-residue truncated protein expected to be processed at an PEXEL motif (RxLxE/D/Q at residues 48–52); importantly, the truncated protein lacks a lysine-rich domain (residues 119–164) previously implicated in erythrocyte aggregation (8). We successfully generated viable knockouts, confirming that PfGARP is not essential for *in vitro* survival and replication. This knockout is designated as PfGARP KO (NIH) in this study. The limiting dilution cloning yielded the GARP-KO3D7 and GARP-KODd2 clones, with confirmed replacement of the native locus (see also Fig. S1). Brief experimental protocol is outlined below.

The *P*. *falciparum* laboratory lines were cultivated in commercially obtained O+ human erythrocytes (UVA blood bank) and RPMI 1640 medium supplemented with 50 mg/L hypoxanthine, 25 mM HEPES, 0.23% NaHCO3, 0.5% NZ Microbiology BSA (MP Biomedicals) and drug selection as indicated; cultures were maintained at 5% hematocrit and 37 °C under 5% O_2_, 5% CO_2_, 90% N_2_. A 616 bp *pfgarp*-ko homology cassette was constructed by DNA synthesis and cloned into pL6-NanoLuc-BSD at unique XbaI and NcoI sites (56). Annealed sgRNA oligos were cloned into the pL6 plasmid that was linearized at tandem BtgZI sites by In-Fusion HD cloning (Takara Bio) to produce the pL7-garpKO-BSD transfection plasmid. The targeted construct was confirmed by restriction digest analysis and DNA sequencing.

For DNA transfection and limiting dilution cloning, human erythrocytes were loaded with required plasmids at 50 μg each in a 2 mm-gap cuvette using the Gene Pulser Xcell system (Bio-Rad), washed, and diluted to a 1.75% hematocrit (Hct) final concentration. Trophozoite-stage parasites were enriched by Percoll-sorbitol centrifugation and added to a final 2% parasitemia. After cultivation for 2 days, fresh erythrocytes were added to a final 5% Hct with initiation of drug selection. Transfection of 3D7 utilized a two-plasmid transfection strategy (57) with pL7-garpKO-BSD and pUF1-Cas9 to provide SpCas9, followed by selection with 2.5 μg/ml Blasticidin S (InvivoGen) and 1.5 μM DSM1 (BEI Resources). The knockout in the Dd2 background used a previously generated PfCD clone that expresses SpCas9, permitting single-plasmid transfection with pL7-garpKO-BSD and Blasticidin S selection (56). After parasite outgrowth, limiting dilution cloning and PCR confirmation, drug selection was removed for cultivation of knockout clones in drug free media (58). The plasmid map of GARP-KO-pL7-MT6-Nluc-BSD.gbk (Circular/6328 bp) construct is shown (see also Fig. S1).

### Dot blot Western blotting

Nitrocellulose membrane was cut into small squares and 2 μl of peptides or recombinant protein at concentrations of 1.0 μg/ml were spotted and air dried. Recombinant proteins TRX, TRX-PfGARP-M, and TRX-PfGARP-M2 were immobilized. Membranes were washed in Tris-buffered saline with 0.05% Tween-20 (TBST) and then blocked with 5% BSA in TBST for one hour at room temperature. Immobilized peptides and proteins were incubated with either M2K5 or M1P6 peptides (8). Streptavidin-AP antibody (1:5,000, Abcam) was diluted in blocking buffer for 1 h at room temperature. Membranes were developed with Bio-Rad Immune Star AP developing kit and visualized on a ChemiDoc XRS + Imaging System (Bio-Rad) with autoexposure settings.

### Co-immunoprecipitation and unbiased mass spectrometry

For co-immunoprecipitation (Co-IP), high parasitemia cultures of wild-type 3D7, V5-tagged PfGARP 3D7, PIESP2 knockout, and two independent PfGARP knockout (NIH and HMS) parasites were each passed through a magnetic column to isolate mature-stage parasites using MACS (Miltenyi Biotec). Schizont pellets were resuspended in radioimmunoprecipitation assay (RIPA) lysis and extraction buffer (Thermo Scientific, 50 mM Tris-HCl pH 8.0, 150 mM NaCl, 1% NP-40, 0.5% sodium deoxycholate, 0.1% SDS, and Pierce protease inhibitor tablets without EDTA) on ice for 30 min and clarified at 14,000*g* for 5 min at 4 °C. Magnetic Dynabeads Protein G (Invitrogen, catalog # 10003D) were washed twice with RIPA lysis buffer and added to lysed parasite samples for one hour rotating at 4 °C to preclear lysates. 5.0 μg of GM7mAb, mAb7899, or anti-V5 tag mAb (Bio-Rad SV5-Pk1) was added to pre-cleared lysate for one hour at 4 °C with rotary agitation. Magnetic beads used for preclearing were removed using a DynaMag-2 magnet (Invitrogen) and discarded. During antibody incubation, slurries of magnetic DynaBeads Protein G or Pierce Protein G Agarose (Thermo Scientific) were washed with PBS and blocked with 3% BSA in PBS for one hour at 4 °C. 50 μl of cleaned and blocked magnetic or agarose beads were added to the antibody-lysate mixture and incubated for one hour at 4 °C with gentle agitation. Bead-IgG-antigen complexes were washed six times with RIPA lysis buffer and five times with 50 mM ammonium bicarbonate, pH 7.8, before being analyzed at the Taplin Mass Spectrometry (MS) Facility at Harvard Medical School. Immunoprecipitation and subsequent mass spectrometry analyses were repeated for a total of two biologically independent experiments.

### Immunofluorescence microscopy

For cell permeable immunofluorescence analysis (IFA), thin blood smears of wild-type 3D7, two independent PfGARP knockouts (NIH and HMS), and PIESP2 knockout parasite cultures were prepared, fixed and permeabilized with ice-cold (−20 °C) 100% methanol for 15 to 45 min. Smears were blocked with 3% bovine serum albumin (BSA) in PBS for 30 min and washed twice with PBST (0.05% Tween-20). Anti-PfEMP1 pAb (1:100), GM7mAb (1:2000), anti-band 3 pAb (1:50), anti-KAHRP pAb (1:50), and anti-MSP1 pAb (1:50) were diluted in blocking buffer and incubated with slides at room temperature for one hour. Smears were washed three times with PBST and incubated with anti-mouse IgG conjugated with Alexa Fluor 488 (1:1000 dilution, Invitrogen) or anti-rabbit IgG conjugated with Alexa Fluor 546 (1:1000 dilution, Invitrogen) for signal detection.

To label cell surface antigens under non-permeabilizing conditions, cells were fixed in suspension for one hour at room temperature in PBS containing 4% paraformaldehyde and then washed three times with PBS. Non-permeabilized fixed cells were incubated with GM7mAb (1:2000) or mAb7899 (1:1000) in 3% BSA in PBS for one hour, washed three times in PBS, and then incubated for one hour with anti-mouse IgG conjugated with Alexa Fluor 488 (1:1000 dilution, Invitrogen) diluted in 3% BSA in PBS. Finally, cells were washed three times in PBS and mounted on slides for imaging. All slides were mounted with ProLong Diamond Antifade mountant with 4′,6-diamidino-2-phenylindole (DAPI) (Invitrogen). Cells were imaged using a Nikon Eclipse TE2000-E microscope equipped with a 100X oil-immersion objective and an exposure of 300 milliseconds. Immunofluorescence assays were also performed using control IgG, uninfected RBCs, and secondary antibodies only, as described above.

For quantification of surface-associated fluorescence in non-permeabilized infected erythrocytes (Fig. 7, *C* and *D*; Scale bar, 5 μm), individual iRBCs were manually outlined in ImageJ based on visible fluorescence signal. Background fluorescence was measured from cell-free regions within the same image. For each cell, corrected mean fluorescence intensity (MFI) was calculated by subtracting the corresponding background signal. Five representative cells per group were analyzed and plotted. Data are presented as mean ± SD. Statistical analysis was performed using Kruskal–Wallis test followed by Dunn’s for pairwise multiple comparisons; ns, not significant.

### Confocal immunofluorescence analysis (IFA)

The MACS-purified and synchronized iRBCs at 3 to 5% parasitemia were used for confocal microscopy. Thin blood smears of wild-type (WT) and PfGARP knockout (PfGARP KO) parasites were air-dried and fixed at room temperature with 100% acetone for two minutes. Smears were blocked with 3% bovine serum albumin (BSA) in PBS for 30 min and washed with PBS containing 0.05% Tween-20 (PBST). Slides were sequentially incubated with mouse GM7mAb (1:2000) and anti-SBP1 (1:500) rabbit polyclonal primary antibodies for one hour each at room temperature, with three washes in PBS between steps. Alexa Fluor 488 anti-mouse and Alexa Fluor 568 anti-rabbit secondary antibodies (1:1000) were applied for one hour in the dark. Nuclear staining was performed using Hoechst 33,342 (1:8000) prior to mounting. Slides were mounted with SlowFade Gold antifade reagent and imaged on a Zeiss LSM 880 confocal microscope equipped with a 63 × /1.4 NA oil-immersion objective. Image acquisition settings were kept identical between WT and KO samples.

### Immunogold-transmission electron microscopy

Magnetically purified schizonts were fixed on a cushion of PBS containing 4% paraformaldehyde (PFA) and 0.01% glutaraldehyde (GA) for three minutes before centrifugation at 3000 rpm. Supernatant was discarded, and fresh fixative was added for one hour at room temperature before being replaced with PBS. Parasite pellets were infiltrated with 2.3M sucrose in PBS containing 0.2M glycine for 15 min. Frozen samples were sectioned at −120 °C and ∼80 nm sections were picked up on a drop of 2.3M sucrose with a small amount of 2% methyl cellulose added (9:1) and transferred to formvar-carbon coated copper grids. Immunolabeling was carried out at room temperature on a piece of parafilm. Grids were gloated on drops of 1% BSA for 10 min to block nonspecific labeling before transferring to five uL drops of GM7mAb diluted in 1% BSA in PBS for 30 min at room temperature. Primary antibody was washed with four drops of PBS (total 10 min) before incubation with 10 nm Protein A-gold for 20 min and were washed with two drops of PBS followed by four drops of water (total 15 min). The labeled sections were contrasted and embedded in methyl cellulose by floating the grids on a mixture of 0.3% uranyl acetate in 2% methyl cellulose for 5 min and excess liquid was blotted off on a filter paper. For transmission electron microscopy (TEM), the grids were examined in a JEOL 1200EX transmission electron microscope and images were recorded with an AMT 2k CCD camera.

### Immunoblot analysis

High parasitemia cultures of mature-stage wild-type 3D7, two independent PfGARP knockouts (NIH and HMS), and PIESP2 knockout parasites were purified on Miltenyi Biotech LS columns (MACS) and resuspended in distilled water (1:25 v/v) to lyse the cells. For RIPA extraction of the schizonts pellet, parasites were resuspended in RIPA buffer with protease inhibitors (Pierce Protease Tablets catalog # PIA32955; Thermo Scientific) on ice for 10 min. Alternatively, parasite pellets were prepared by lysis with 0.15% saponin in PBS on ice for 10 min, followed by a wash with fresh PBS. Schizonts were pelleted at 3000*g* for 5 min before solubilization in sample buffer with reducing agent (BME). Samples were heated at 95 °C for 10 min before analysis by 12% or 4 to 20% gradient SDS-PAGE separation. Proteins were transferred to nitrocellulose membranes and blocked with either 5% milk in PBST or 3% BSA in PBST for one hour. Membranes were probed with GM7mAb (1:50,000) and detected by anti-mouse IgG-HRP. An anti-*Plasmodium* aldolase rabbit polyclonal antibody (ICL Lab, catalog # RPVA-55A) diluted at 1:1000 was used to normalize loading of parasite proteins and was detected by an anti-rabbit IgG-HRP conjugated antibody. For enhanced chemiluminescent (ECL) detection, membranes were developed with SuperSignal West Pico PLUS chemiluminescent substrate (Thermo Scientific) and visualized on a ChemiDoc XRS + Imaging System (Bio-Rad) with Image Lab Software using autoexposure settings. For multiplex fluorescence immunoblots of wild-type 3D7 and PfGARP KO parasite lysates probed with GM7mAb and Pfaldolase, secondary antibodies (Cell Signaling Technology DyLight anti-mouse 800 and anti-rabbit 680) were diluted to 1:25,000 or 1:30,000 in blocking buffer for 45 min in the dark at room temperature. Chameleon Duo pre-stained protein ladder (LI-COR Biosciences) was used to visualize protein bands from 8 to 260 kDa. Imaging was performed with a LI-COR Odyssey CLx for near-infrared detection using autoexposure settings.

### Isolation of knobs from malaria-infected erythrocytes

Wild-type 3D7-infected erythrocyte knobs were isolated by a method described previously (29). Trophozoite and schizont-infected erythrocytes were magnetically separated from uninfected RBCs using MACs. Recovered schizont pellets were washed with RPMI-1640 and then resuspended in RBC membranes (ghosts) lysis buffer (5 mM sodium phosphate and 0.5 mM EDTA, pH 8.0) and incubated on ice for five minutes. Infected and uninfected erythrocyte ghosts were centrifuged at 12,000*g* for 10 min at 4 °C and washed with five volumes of lysis buffer until the ghosts’ pellet turned white in color. Ghosts were then solubilized in 1.5% Triton X-100 for 30 min on ice. Detergent solubilized ghosts were layered on a sucrose step gradient consisting of 75%, 60%, and 10% sucrose layers and centrifuged at 17,300*g* for 15 min. Uninfected RBC ghosts, infected RBC ghosts, and detergent-resistant pellets were analyzed by SDS-PAGE followed by immunoblotting with anti-KAHRP rabbit pAb and GM7mAb (1:50,000). Blots were developed with SuperSignal West Pico PLUS chemiluminescent substrate (Thermo Scientific) and signal was detected using a ChemiDoc XRS + Imaging System (Bio-Rad) using autoexposure settings.

### Expression and purification of recombinant PIESP2

The codon optimized ectodomain of PIESP2 (AA 30–354) was synthesized and cloned into the pET-32a(+) vector using the KpnI and XhoI restriction sites by GenScript. *E*. *coli* BL21 (DE3) expression cells (New England Biolabs; NEB) were transformed with the recombinant PIESP2 plasmid. Transformants were grown in LB supplemented with 100 μg/ml ampicillin, at 37 °C, until an optical density at 600 nm (OD_600_) of 0.5 to 0.7 was reached. Cultures were induced with 0.3 mM IPTG and incubated overnight at 18 °C. Bacterial cultures were centrifuged at 6,000*g* for 10 min at 4 °C and pelleted bacteria was resuspended in 500 mM NaCl, 20 mM Imidazole (OmniPure), 0.5% Triton X-100, DNase I (5 μg/ml), pH 7.4 with protease inhibitor cocktail (Thermo Scientific) and incubated on ice for 30 min before sonication. Lysates were clarified at 12,000*g* for 45 min at 4 °C. Protein purification was achieved through a two-step process using nickel affinity followed by anion exchange chromatography using an ÄKTA FPLC (GE Healthcare). Clarified lysate was applied to an equilibrated HisTrap HP (5 ml) column (Cytiva), washed with lysis buffer, and eluted with a continuous gradient using 500 mM imidazole. Eluted Ni-NTA fractions were analyzed by SDS-PAGE and Coomassie staining and fractions containing TRX-PIESP2 were pooled and diluted with ion-exchange loading buffer (50 mM sodium phosphate dibasic, 50 mM NaCl, pH 7.5) before loading on to an equilibrated HiTrap Q XL (5 ml) column (Cytiva). TRX-PIESP2 was eluted with a continuous salt gradient (50 mM sodium phosphate dibasic, 1.0 M NaCl, pH 7.5). Anion exchange purification eluted fractions were analyzed by SDS-PAGE and Coomassie staining to confirm desired protein purity. Purified TRX-PIESP2 was dialyzed over night at 4 °C in PBS and diluted to a desired concentration before freezing single-use aliquots at −70 °C. TRX-PIESP2’s reactivity with GM7mAb was tested and confirmed using ELISA (enzyme-linked immunosorbent assay) and immunoblotting.

### ELISA: detection of antibody response against PIESP2 in a malaria-endemic region

Immulon 2HB 96-well plates (Thermo Scientific) were saturated with 50 μl (50 ng/ml) recombinant TRX-PIESP2 in PBS overnight at 4 °C. Plates were washed with blocking buffer (3% BSA and 1% gelatin in PBST) to remove unbound excess protein and blocked with fresh blocking buffer for one hour at room temperature. Excess blocking buffer was washed with PBST. Human plasma was diluted 1:400 in PBST and incubated on the plate for one hour at room temperature. Uninfected human serum (H1), PBS, and *Babesia microti* infected patient sera (B1 and B2) were used as negative controls in addition to blocking buffer blanks. Unbound plasma was removed from the wells by washing five times with PBST for five minutes each wash. A 1:4000 dilution of HRP-conjugated anti-human IgG (Jackson ImmunoResearch Laboratories, Inc.) in PBST was incubated in the wells for one hour at room temperature. Wells were washed five times with PBST for five minutes each before colorimetric detection. TMB substrate was added to let the color develop. After 10 minutes, the reaction was quenched with 1.0 M HCl and the absorbance at 450 nm was measured using a VersaMax microplate reader (Molecular Devices). Plasma samples were tested in triplicate for quality assurance and statistical analysis. A reactivity cutoff was statistically determined (ELISA Data Analysis. Bioreba) as follows: (x + 3s)•1.1 where x and s are the mean optical density value and standard deviation for the control plasma samples. Any absorbance value above this cutoff threshold value was considered a positive sample hit.

To quantify PIESP2 antibodies by a peptide-based ELISA, the Immulon plates were coated with 100 μl of streptavidin at 5 μg/ml in PBS overnight at 4 °C. After washing, wells were coated with 100 μl of PIESP2 peptide (PSP-25) at 2.0 μg/ml overnight in PBS. Wells were washed to remove unbound PIESP2 peptide and blocked with 250 μl of blocking buffer (3% BSA in PBST) for one hour at room temperature. Wells were washed with PBST to remove excess blocking buffer. Plasma samples diluted 1:3000 in 3% BSA-PBST (100 μl per well) were incubated for one hour at room temperature. Unbound plasma was removed by washing five times with PBST (100 μl each well) for 5 min. Anti-human secondary antibody conjugated to HRP was diluted 1:4000 in PBST (100 μl) was incubated for one hour at room temperature. Plates were washed five times with PBST and colorimetric signal was measured as described above. A cutoff value was determined for each plate.

### Live cell flow cytometry

Knobby wild-type 3D7, PfGARP knockout, and PIESP2 knockout parasites were prepared for analysis by flow cytometry without fixation or permeabilization. Approximately 5 × 10^6^ erythrocytes at 1 to 5% parasitemia were stained with GM7mAb (1:50) in PBS containing 2% fetal bovine serum (FBS) for 30 min at room temperature. Cells were washed three times with PBS for 5 min at 500*g*. Bound antibody was detected using Alexa Fluor 488-conjugated anti-mouse IgG (1:1000) in PBS containing 2% fetal bovine serum (FBS) for 30 min in the dark at room temperature. Cells were washed three times with PBS for 5 min. Parasitized erythrocytes were stained with Hoechst 33,342 dye (Thermo Fisher Scientific) dissolved in PBS (2 μm). Single-staining controls, unstained/autofluorescence controls, and uninfected RBC controls were used in addition to a negative control mAb7899 (1:50 dilution) and an isotype control mouse IgG_3_ (1:50 dilution) (SouthernBiotech; cat. # 0105–01). Signal was acquired on a Bigfoot Spectral Cell Sorter (Invitrogen) with Sasquatch (SQ) software using the UV laser (349 nm) to detect Hoechst 33,342 and the blue laser (488 nm) for AF488. The Invitrogen Bigfoot Cell Sorter is equipped with integrated biocontainment and aerosol management for biosafety permitting experiments using live and unfixed cells. The FlowJo software (Version 10) was used to analyze and gate 500,000 events from each sample.

### Mapping of the GM7mAb epitope within PIESP2

To identify the precise GM7mAb epitope within PIESP2, the extracellular surface exposed segment of PIESP2 (AA 30–354) was divided into two segments (AA 30–114 and AA 115–354 amino acids; Fig. 9*B*). The codon optimized cDNA of PIESP2 was synthesized and cloned in KpnI/XhoI sites of pET-32a(+) plasmid by GenScript. Smaller fragments derived from PIESP2 (AA 115–154; 155–228; 229–354) (Fig. 9*B*) were PCR amplified using primers containing KpnI adaptor (forward primers) and XhoI adaptor (reverse primers) using PIESP2 (30–354 aa)-pET-32a (+) plasmid as a template. Following KpnI and XhoI digestion, the fragments were cloned into pET-32a (+) plasmid at KpnI/XhoI sites. For protein expression studies, the *E*. *coli* BL21 (DE3) were transformed with corresponding plasmids, expression was induced by IPTG (0.1 mM), and the bacterial cells were collected by centrifugation and lysed directly in SDS-PAGE sample buffer. Proteins were resolved in SDS-PAGE followed by transfer onto nitrocellulose membrane. Expression of each of the TRX-PIESP2 protein construct was observed as a prominent band in the ponceau S-stained membrane. The same membrane was used for Western blot detection with GM7mAb (1:5000). The PIESP2 segment (AA 155–228) recognized by GM7mAb is shown in red.

### Characterization of GM7mAb specificity

The technical details of the development of GM7 monoclonal antibody were reported as supplemental data in our previous publication (8). Briefly, recombinant His-PfGARP-M expressed in bacteria was purified by Mono Q ion exchange FPLC system, and mouse hybridomas were sub-cloned to collect the IgG3 monoclonal antibody termed GM7mAb. PfGARP-M consists of 75-amino-acid segment containing 21 glutamic acid (E) residues and 27 lysine (K) residues with a predicted isoelectric point (pI) of 9.1. The GM7mAb recognized discrete repeat motifs enriched in both E and K residues and its immunoreactivity was mapped to the major M1P6 peptide in PfGARP as confirmed by competitive ELISA and Western blotting (8).

The initial characterization of GM7mAb by immunofluorescence assay (IFA) included uninfected erythrocytes as a negative control. To rule out the possibility that GM7mAb may detect reticulocytes within the same flow cytometric gate, flow cytometry was performed on uninfected RBCs demonstrating the absence of GM7mAb staining within this population (Fig. S3). These findings exclude the recognition of residual reticulocytes as well as any glycans by GM7mAb on the surface of host erythrocytes. Similarly, our previous IFA studies demonstrated that GM7mAb did not detect any signal on RBCs infected with ring-stage parasites (8). These observations provide strong evidence that GM7mAb does not recognize any negatively charged structures on the surface of either uninfected or ring-infected host erythrocytes.

The immunoblot analysis of the PIESP2 knockout parasite line showed no detectable GM7mAb-reactive protein species (Fig. 4*F*, middle panel). Furthermore, our unbiased immunoprecipitation and mass spectrometry analyses failed to identify additional *P*. *falciparum* proteins that could account for the observed surface staining or serve as alternative knob-associated targets of GM7mAb. Together, these findings support the conclusion that the GM7mAb signal is attributable to PfGARP and/or PIESP2 rather than to an unrelated knob-associated antigen.

### Static adhesion of IEs (iRBCs) to human cerebral microvascular endothelial cells (HBEC-5i-CRL-3245 line. ATCC)

To measure adhesion of IEs to human brain endothelial cells, *in vitro* cultures of wild-type 3D7, PfGARP knockout, and PIESP2 knockout parasites were tested for static binding to HBEC-5i. All three parasite lines were subject to five rounds of selection against HBEC-5i. Assays were performed in 24-well tissue culture plates coated with 0.1% gelatin and seeded ∼48 h with 80 to 90% confluence. Adherent HBEC-5i were washed twice with 0.5 ml of pre-warmed incomplete DMEM medium. Synchronized late-stage IEs resuspended in pre-warmed binding medium (incomplete DMEM medium with 1% BSA) were added to duplicate wells (1 × 10^7^/ml, 200 μl per well) and incubated at 37 °C in 5% CO_2_ for 75 min with gentle resuspension at the 30- and 60-min marks. After incubation, the medium was aspirated to remove any unbound cells and wells were washed four times in the binding medium with gentle rocking. The presence of unbound cells was monitored using an inverted Nikon microscope and, if needed, washes were repeated until no unbound IEs remained. Bound cells were fixed for 30 min using 200 μl of 2% glutaraldehyde per well and washed with an equal volume of PBS. Wells were stained with 5% Wright-Giemsa for 10 min, washed with water and air-dried before quantification. To measure binding, sample wells were masked and the number of IEs bound to at least 100 HBEC-5i were assessed in 5 to 10 random fields by two blinded microscopists. To test the ability of different antibodies to block binding of IEs to HBEC-5i cells, an inhibition assay was performed in the same 24-well plate as the static adhesion under normal conditions. Prior to incubation with HBEC-5i, wild-type 3D7, PfGARP knockout, and PIESP2 knockout infected erythrocytes (1 × 10^7^/ml) were incubated with GM7mAb (100 μg/ml), mAb7899 (100 μg/ml), and a mouse IgG3 isotype (100 μg/ml) at 37 °C for 30 min. The IEs were washed twice with the binding medium followed by incubation, washing and quantification of binding as outlined above.

### Freeze-fracture electron microscopy

The distribution of intramembrane particles (IMP), mainly composed of band 3, in *P*. *falciparum-infected* human erythrocytes by freeze-fracture EM was performed by Laura Derick at St Elizabeth’s Medical Center. The samples were prepared by Dr Chishti from purified knobby 3D7 parasite-infected erythrocytes and fractured in a freeze-etch unit (model BAF 400D; Balzers, Hudson, NH) under the supervision of Dr David (Shih-Chun) Liu. The experimental details are described in the relevant publications (59, 60, 61).

### Data availability

All data supporting the findings of this study are contained within the published article and its supporting information.

## Supporting information

This article contains supporting information.

## Conflict of interest

The authors declare that they have no conflicts of interest with the contents of this article.
