# Supplementary figures and images for: Epitope mirroring between the malaria surface proteins PfGARP and PIESP2 identifies a knob-associated complex in infected erythrocytes
Source: J Biol Chem. 2026 Jun 23;302(8):113291. doi: 10.1016/j.jbc.2026.113291 (PMC13400357; doi:10.1016/j.jbc.2026.113291)

Figure S1

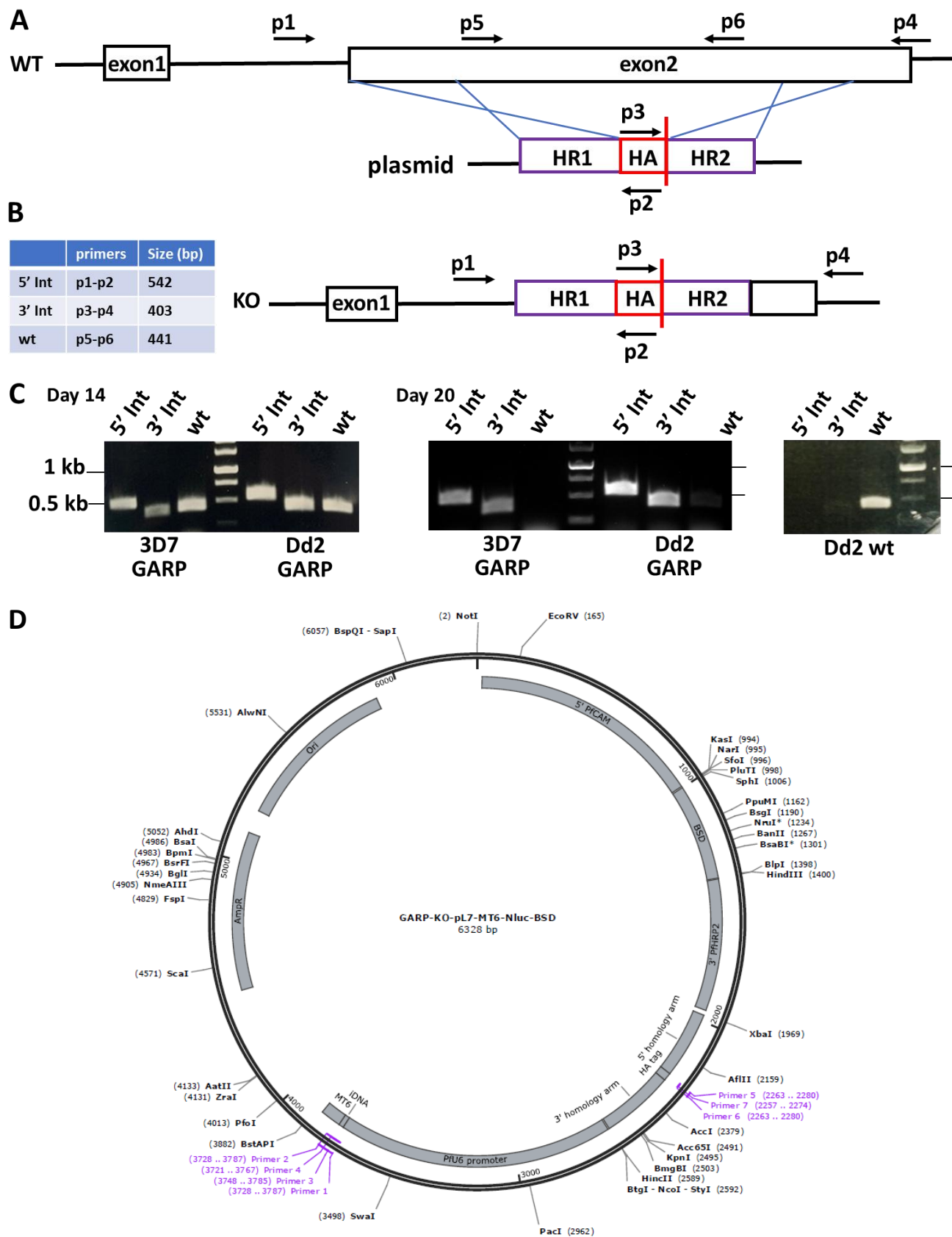

Supplement: Fig. S1 [file mmc1.pdf]

**Figure S2**

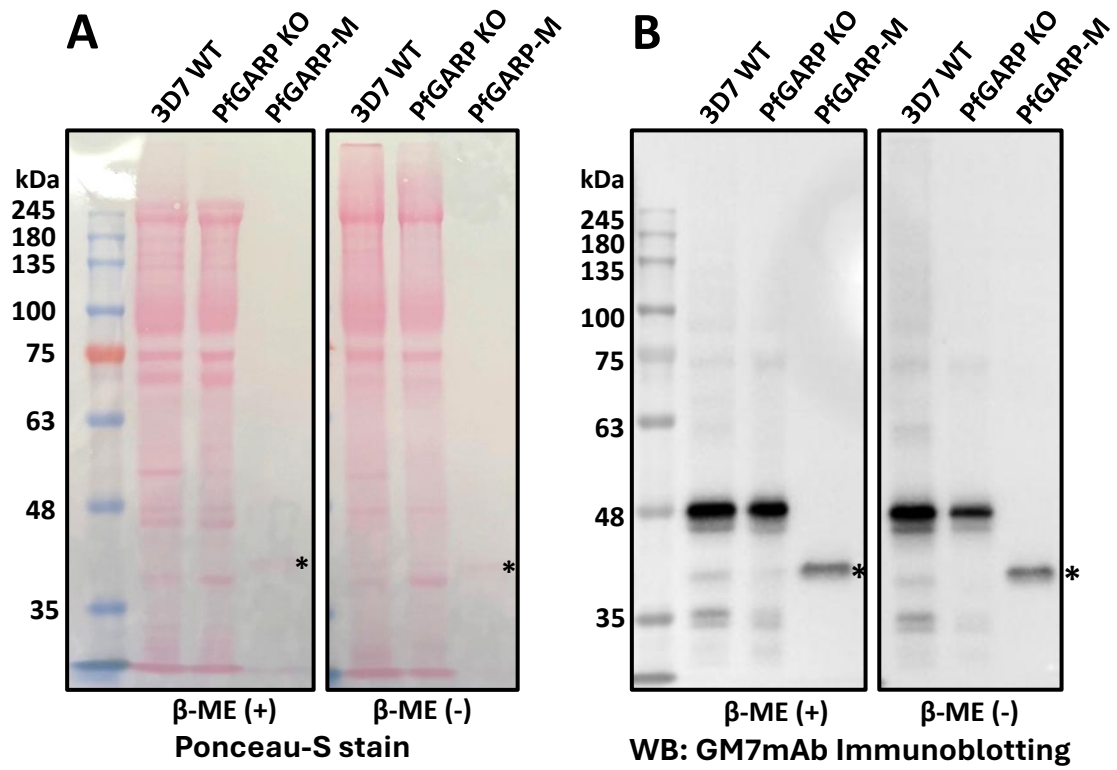

Supplement: Fig. S2 [file mmc2.pdf]

**Figure S3**

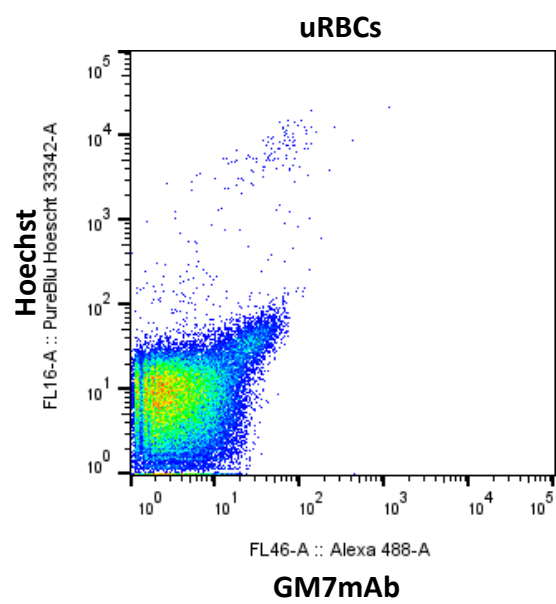

Supplement: Fig. S3 [file mmc3.pdf]

**Figure S4**

**Anti-SBP1 pAb (Red; Alexa 568):**  
**GM7mAb (Green; Alexa 488)**

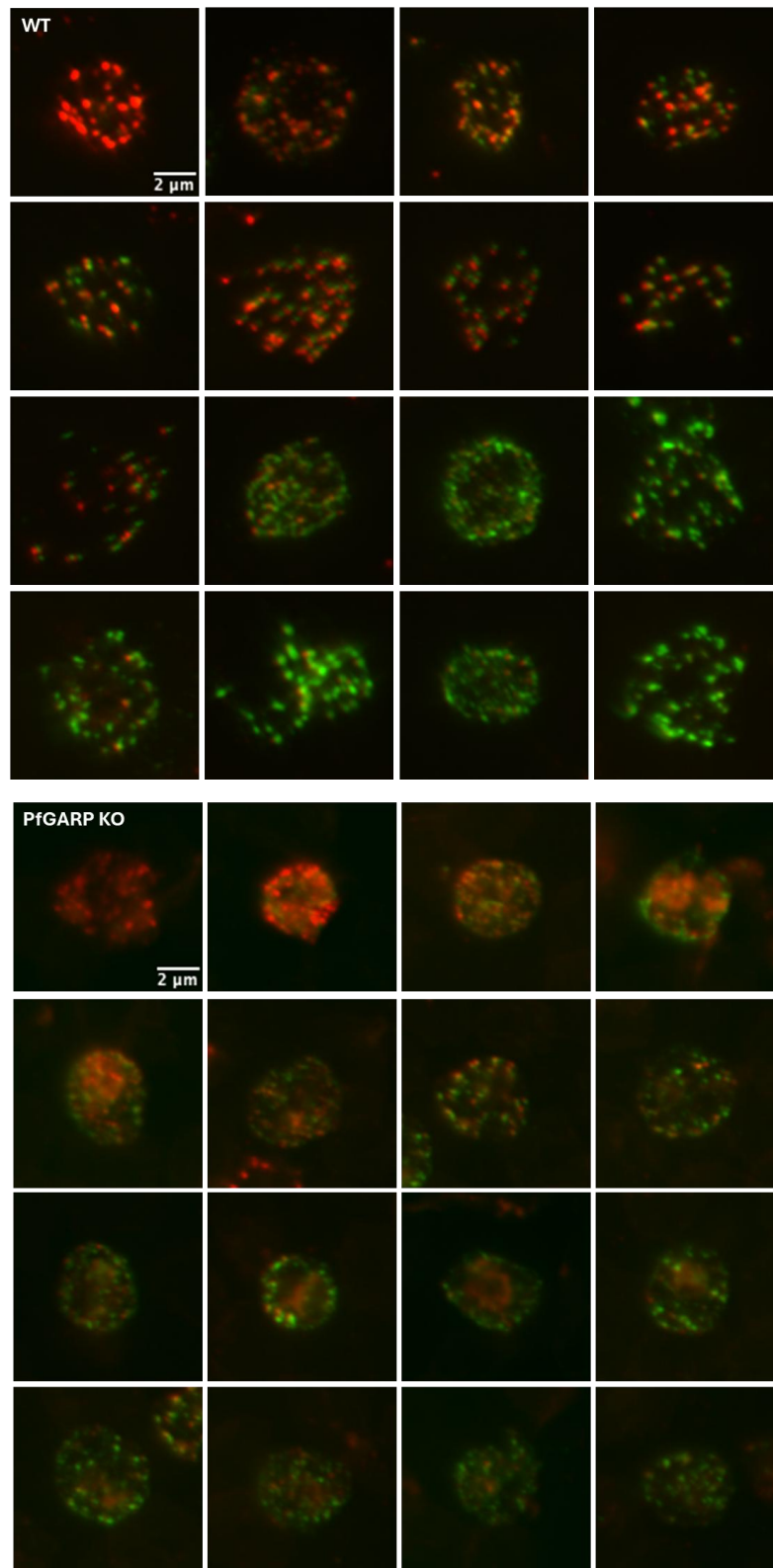

Supplement: Fig. S4 [file mmc4.pdf]

Figure S5

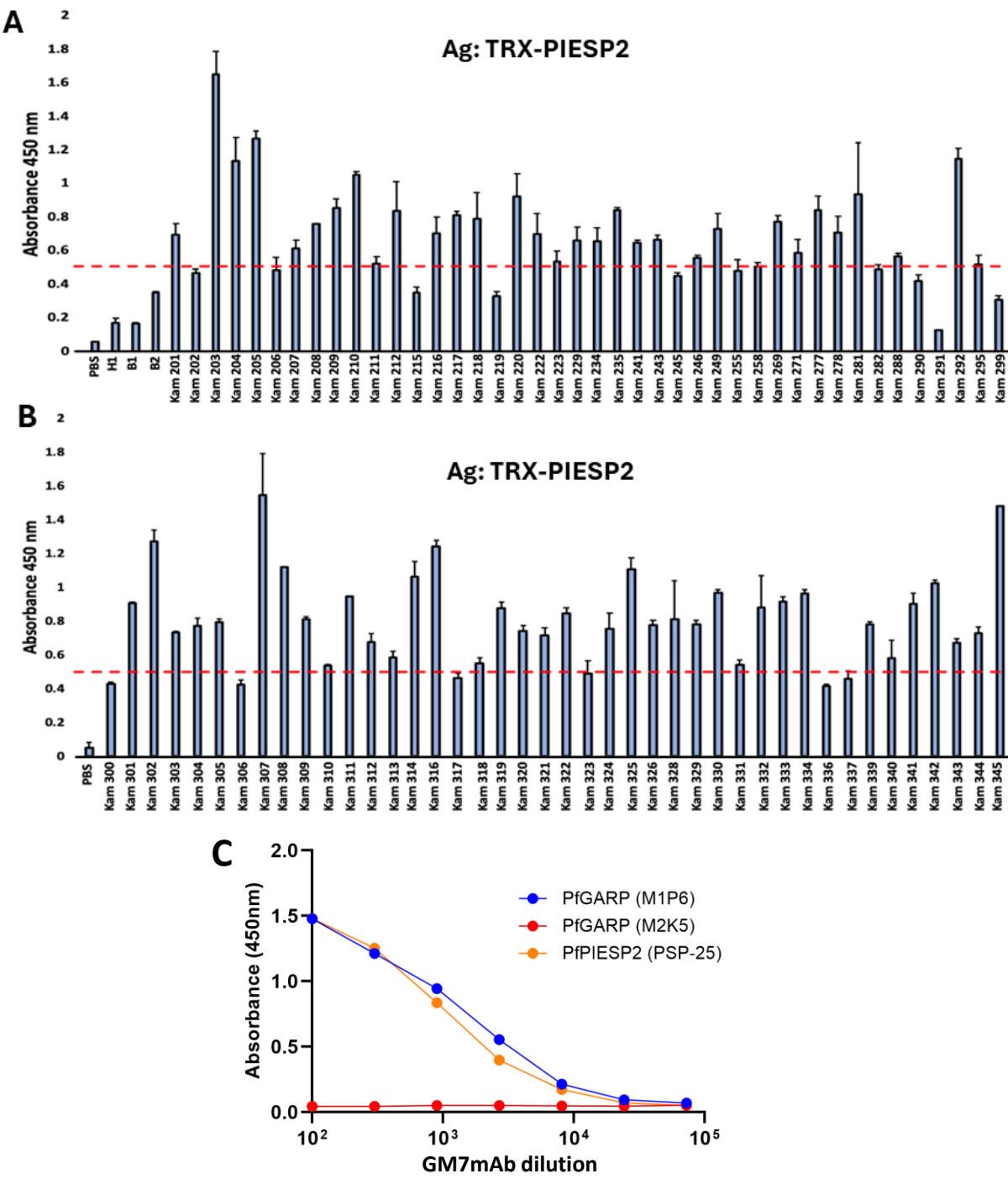

Supplement: Fig. S5 [file mmc5.pdf]
