# Supplementary material for: Epitope mirroring between the malaria surface proteins PfGARP and PIESP2 identifies a knob-associated complex in infected erythrocytes
Source: J Biol Chem. 2026 Jun 23;302(8):113291. doi: 10.1016/j.jbc.2026.113291 (PMC13400357; doi:10.1016/j.jbc.2026.113291)
Supplement: Legend Fig. S1 [file mmc6.docx]

**Figure S1: Construction and validation of PfGARP knockout parasite line (NIH).** **(A)** Schematic of *pfgarp*-ko homology cassette representation of wild-type PfGARP gene. **(B)** PfGARP gene knockout validation by selected primers. **(C)** PCR analysis of corresponding segments. **(D)** Map of GARP-KO-pL7-MT6-Nluc-BSD.gbk (Circular/6328 bp) construct. Additional details about the construct are provided in the Methods section.
