# Supplementary material for: Epitope mirroring between the malaria surface proteins PfGARP and PIESP2 identifies a knob-associated complex in infected erythrocytes
Source: J Biol Chem. 2026 Jun 23;302(8):113291. doi: 10.1016/j.jbc.2026.113291 (PMC13400357; doi:10.1016/j.jbc.2026.113291)
Supplement: Legend Fig. S2 [file mmc7.docx]

**Figure S2: Effect of reducing and non-reducing conditions on GM7mAb reactivity in parasite-infected RBC membrane proteins. (A)** Ghosts were prepared from iRBCs derived from wild-type 3D7 and PfGARP knockout lines. Recombinant PfGARP-M was included as positive control. Ghosts were solubilized in an equal volume of 2 x SDS-PAGE sample buffer in presence and absence of β-mercaptoethanol (β-ME). Samples were analyzed by 10% acrylamide SDS-PAGE. Ponceau-S staining (nitrocellulose membrane) shows equal loading and intact membrane proteins. **(B)** Immunoblotting was performed with GM7mAb. The 48 kDa band corresponding to PIESP2 was detected in both wild-type 3D7 and PfGARP knockout cells. While recombinant PfGARP-M was detected under both reducing and non-reducing conditions, no ~100 kDa band corresponding to full length endogenous PfGARP was detected in the wild-type 3D7 cells.
