# Supplementary material for: Epitope mirroring between the malaria surface proteins PfGARP and PIESP2 identifies a knob-associated complex in infected erythrocytes
Source: J Biol Chem. 2026 Jun 23;302(8):113291. doi: 10.1016/j.jbc.2026.113291 (PMC13400357; doi:10.1016/j.jbc.2026.113291)
Supplement: Legend Fig. S — 3 [file mmc8.docx]

**Figure S3: Flow cytometry analysis of uninfected RBCs.** Flow cytometry was performed on unfixed human RBCs without parasite infection. No GM7 mAb positive signal was detected in uninfected RBCs indicating that GM7 mAb does not recognize negatively charged structures on the surface of uninfected cells.
