# Supplementary material for: Epitope mirroring between the malaria surface proteins PfGARP and PIESP2 identifies a knob-associated complex in infected erythrocytes
Source: J Biol Chem. 2026 Jun 23;302(8):113291. doi: 10.1016/j.jbc.2026.113291 (PMC13400357; doi:10.1016/j.jbc.2026.113291)
Supplement: Legend Fig. S — 4 [file mmc9.docx]

**Figure S4: Comparison of IFA signals between GM7mAb and anti-SBP1 pAb antibody in wild-type 3D7 and PfGARP knockout infected erythrocytes.** The upper panels display representative montage images of wild-type 3D7 iRBCs, whereas the lower panels display representative montage images of PfGARP knockout iRBCs. These data suggest that GM7mAb signal is mostly adjacent to SBP1 (Figure 8D). iRBCs were air-dried and fixed at room temperature with 100% acetone for two minutes. For additional details, refer to the legend in Figure 8D, as well as the Methods and Results sections. Scale bar: 2 μm.
