# Supplementary material for: Epitope mirroring between the malaria surface proteins PfGARP and PIESP2 identifies a knob-associated complex in infected erythrocytes
Source: J Biol Chem. 2026 Jun 23;302(8):113291. doi: 10.1016/j.jbc.2026.113291 (PMC13400357; doi:10.1016/j.jbc.2026.113291)
Supplement: Legend Fig. S — 5 [file mmc10.docx]

**Figure S5: Identification of natural antibodies in human plasma samples using TRX-PIESP2 fusion protein (Panels A, B) and validation of PSP-25 peptide for ELISA (C).** Recombinant TRX-PIESP2 fusion protein (30-354 amino acids; Figure 4A, B) was coated on ELISA plates and screened with plasma samples from human subjects as described in the Results section. PBS (phosphate buffered saline), H1 (uninfected human plasma), and B1/B2 (Plasma samples from *Babesia microti* infected patients) were used as negative controls. Of 84 individuals screened, a positive identification rate of 74% was observed (A, B). A cutoff value for positive identification was ~0.54 absorbance. Additional details are included in the Methods and Results sections. Validation of the PIESP2 peptide (PSP-25) by ELISA (C). The positive control PfGARP peptide (M1P60) and the negative control PfGARP peptide (M2K5) were included to assess the reactivity of GM7mAb to PSP-25 derived from PIESP2. Detailed information on PSP‑25 (Figure 9F) and the additional peptides is provided in the Methods and Results sections.
